# Potential Mimicry of Viral and Pancreatic β Cell Antigens Through Non-Spliced and *cis*-Spliced *Zwitter* Epitope Candidates in Type 1 Diabetes

**DOI:** 10.3389/fimmu.2021.656451

**Published:** 2021-04-15

**Authors:** Michele Mishto, Artem Mansurkhodzhaev, Teresa Rodriguez-Calvo, Juliane Liepe

**Affiliations:** ^1^ Centre for Inflammation Biology and Cancer Immunology (CIBCI) & Peter Gorer Department of Immunobiology, King’s College London, London, United Kingdom; ^2^ Francis Crick Institute, London, United Kingdom; ^3^ Max-Planck-Institute for Biophysical Chemistry, Göttingen, Germany; ^4^ Institute of Diabetes Research, Helmholtz Zentrum München, German Research Center for Environmental Health, Munich-Neuherberg, Germany; ^5^ German Center for Diabetes Research (DZD), Neuherberg, Germany

**Keywords:** T1D, antigen presentation, tolerance, mimicry, spliced peptides, virus

## Abstract

Increasing evidence suggests that post-translational peptide splicing can play a role in the immune response under pathological conditions. This seems to be particularly relevant in Type 1 Diabetes (T1D) since post-translationally spliced epitopes derived from T1D-associated antigens have been identified among those peptides bound to Human Leucocyte Antigen (HLA) class I and II complexes. Their immunogenicity has been confirmed through CD4^+^ and CD8^+^ T cell-mediated responses in T1D patients. Spliced peptides theoretically have a large sequence variability. This might increase the frequency of viral-human *zwitter* peptides*, i.e.* peptides that share a complete sequence homology irrespective of whether they originate from human or viral antigens, thereby impinging upon the discrimination between self and non-self antigens by T cells. This might increase the risk of autoimmune responses triggered by viral infections. Since enteroviruses and other viral infections have historically been associated with T1D, we investigated whether *cis*-spliced peptides derived from selected viruses might be able to trigger CD8^+^ T cell-mediated autoimmunity. We computed *in silico* viral-human non-spliced and *cis*-spliced *zwitter* epitope candidates, and prioritized peptide candidates based on: (i) their binding affinity to HLA class I complexes, (ii) human pancreatic β cell and medullary thymic epithelial cell (mTEC) antigens’ mRNA expression, (iii) antigen association with T1D, and (iv) potential hotspot regions in those antigens. Neglecting potential T cell receptor (TCR) degeneracy, no viral-human *zwitter* non-spliced peptide was found to be an optimal candidate to trigger a virus-induced CD8^+^ T cell response against human pancreatic β cells. Conversely, we identified some *zwitter* peptide candidates, which may be produced by proteasome-catalyzed peptide splicing, and might increase the likelihood of pancreatic β cells recognition by virus-specific CD8^+^ T cell clones, therefore promoting β cell destruction in the context of viral infections.

## Introduction

**Figure 1:**
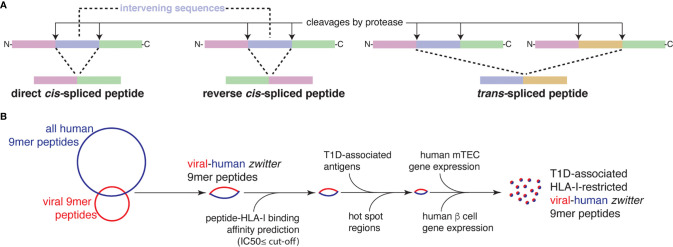
Post-translationally spliced peptides and *in silico* pipeline for the identification of T1D-associated *zwitter* epitope candidates. **(A)** Post-translationally spliced peptides can be formed by: i *cis-*peptide splicing, when the two splice-reactants, *i.e*. the non-contiguous peptide fragments ligated by a protease, derive from the same polypeptide molecule; the ligation can occur in normal order, *i.e.* following the orientation from N- to C-terminus of the parental protein (direct *cis*-spliced peptides), or in the reverse order (reverse *cis*-spliced peptides); ii. *trans*-peptide splicing, when the two splice-reactants originate from two distinct protein molecules or two distinct proteins (6). **(B)** *In silico* pipeline to identify a pool of *zwitter* non-spliced or *cis*-spliced epitope candidates associated with T1D.

Type 1 diabetes (T1D) is an autoimmune disease with a pivotal T cell activity. Indeed, CD4^+^ and CD8^+^ T cell-mediated responses in T1D patients play a prominent role in pancreatic β cell death, and the consequent insulin-dependent disease. CD4^+^ and CD8^+^ T cells recognize fragments (peptidic epitopes) of antigens, which are presented to T cell receptors (TCRs) αβ through Human Leucocyte Antigen class I and class II (HLA-I and -II) complexes. Since autoreactive T cells selectively recognize antigenic epitopes specific for pancreatic β cells, part of the research in T1D sails in uncharted waters to discover neoantigens, epitopes and their presentation mechanisms, which can explain why CD4^+^ and CD8^+^ T cells build an autoreactive immune response in T1D. In the last decade, huge progress in mass spectrometry and bioinformatics has allowed the identification of unconventional antigenic peptides, *i.e*. peptides that could not be directly identified in the human proteome. Cryptic peptides derived from putative non-coding regions, usage of alternative open reading frames, as well as post-translational modifications emerged as a sizeable portion of the peptides that are presented by HLA-I and -II complexes to T cells (1–5). Among them, post-translationally spliced epitopes derived from T1D-associated antigens represent an attractive source of neoantigens. These peptides are produced by fusion of two non-contiguous peptide fragments of either an antigen – i.e.* cis*-spliced peptides – or two distinct antigens, *i.e. trans*-spliced peptides (6) (**Figures 1A, B**). Hybrid insulin peptides (HIPs) identified by Delong and colleagues (7), belong to the latter category. Indeed, they are formed by the fusion of a splice-reactant of insulin, and another derived from other T1D-associated antigens. HIPs are presented by major histocompatibility complexes class II molecules (MHC-II) in nonobese diabetic (NOD) mice and by HLA-II (HLA-DP, HLA-DM, HLA-DOA, HLA-DOB, HLA-DQ, and HLA-DR) in humans (7–9). A handful of known HIPs can trigger a CD4^+^ T cell response both in NOD mice and in T1D patients (7–11). The enzymes (or biochemical reactions) catalyzing their production are not fully understood, although pioneer studies suggest that HIPs or HIPs’ precursors might be produced in β cell’s insulin crinosomes or professional antigen presenting cell (APC)’s lysosomes (9, 12, 13). Their identification, however, is still controversial, and the employment of different mass spectrometry data analysis strategies has led to contradictory results in HIPs’ identification (12, 14).

Many more antigenic *cis*-spliced peptides have been identified in the context of HLA-I antigen processing and presentation (APP) pathway since their first appearance in the literature in 2004 (6, 15, 16). They can target *in vivo* CD8^+^ T cell responses against bacterial antigens, otherwise neglected in a mouse model of *Listeria monocytogenes* infection (17). *Cis*-spliced epitopes derived from either melanoma-associated or T1D-associated antigens are recognized by CD8^+^ T cells in peripheral blood of melanoma patients and T1D patients, respectively (18–20). *Cis*-spliced as well as non-spliced peptides bound to HLA-I complexes are mainly produced by proteasomes (16, 18, 21). During *in vitro* digestions of synthetic polypeptides, proteasomes seem to produce almost as many *cis*-spliced as non-spliced peptides in terms of number of different peptide products (22). However, on average, *cis-*spliced peptides are generated in lower amounts than non-spliced peptides (23, 24), thereby suggesting that peptide splicing is catalyzed by proteasomes less efficiently than peptide hydrolysis. The identification of *cis*-spliced peptides among peptides bound to HLA-I complexes - *i.e*. HLA-I immunopeptidomes – is at least as controversial as that of HIPs; depending on the method used for *cis*-spliced peptide identification, their frequency in HLA-I immunopeptidomes was estimated to range from 1% to 34% (25).

The large variety of *cis*-spliced peptide sequences might have been problematic for models of self/non-self discrimination and tolerance of CD8^+^ T cells (26). Indeed, the vast human and virus *cis*-spliced peptide database might significantly increase the theoretical number of viral-human *zwitter* peptides. *Zwitter* is the German word for hybrid. It is etymologically derived from *zwi*-, “duplex”. For example, in chemistry, a zwitterion is an ion which possesses both positively- and negatively-charged groups. We use the term ‘*zwitter* peptides’ to describe peptides with a sequence that may be generated from viral antigens as well as from human antigens (6, 27). A large number of viral-human *zwitter cis*-spliced peptides might impinge upon CD8^+^ T cell repertoires and the recognition of viral antigens through central and peripheral tolerance. Nevertheless, according to a preliminary *in silico* study, this does not seem to be a frequent occurrence (27). This is in part due to the fact that only a tiny fraction of all theoretical *cis*-spliced peptides is *de facto* produced by proteasomes, as suggested by *in vitro* digestions of synthetic polypeptides with proteasomes measured by mass spectrometry (22).

Nonetheless, even a few viral-human *zwitter* (*cis*-spliced) epitopes may act as the targets of a CD8^+^ T cell autoreactive response, triggered by a viral infection. A growing number of immunological studies suggest that thymic clonal deletion prunes but does not completely eliminate autoreactive CD8^+^ T cells (28–30). Therefore, naïve CD8^+^ T cell clones specific to viral-human *zwitter* epitopes may circulate in the body and be controlled by peripheral tolerance. Central and peripheral tolerance, however, does not completely eliminate the risk that some of these naïve CD8^+^ T cell clones may recognize the cognate epitopes during a viral infection, be primed, proliferate, and then carry out an autoreactive response, for instance during a second or multiple viral infections. This might be the case of T1D, wherein CD8^+^ T cells could recognize viral-human *zwitter* epitopes which are also presented by pancreatic β cells. T1D has been historically associated with viral infections and especially with enteroviruses (EVs), such as Coxsackievirus B1 and B4 (CVB1 and CVB4; **Table S1**) (31). However, to date, the detection of circulating or infiltrating EV‐reactive T cells has been challenging. CD8^+^ T cells predominate in the islets, many of which express high levels of HLA-I molecules at disease onset, likely contributing to exacerbated antigen presentation. EVs are known to infect pancreatic β cells and induce an inflammatory response in the islets (32). Sequence similarity has been previously described through the study of canonical non-spliced epitope candidates, between the 2C non-structural protein (P2C) of CVB4 and the glutamate decarboxylase 2 (GAD2; a.k.a. GAD65) antigen, which is predominantly expressed in pancreatic β cells, although no evidence of cross-recognition by CD8^+^ T cell clones was demonstrated in that report (33). Other members of the Picornaviridae family, such as Parechoviruses (HPeV2), have also been associated to T1D (34). Similarly to EVs, a possible link was described between Rotaviruses – such as Rotavirus C (RVC) - and T1D, because of a potential molecular mimicry between the VP7 protein of a human rotavirus strain and I-A2 (35) and GAD65 (36), although in the latter case the response was limited to CD4^+^ T cells.

Viral and β cell epitopes might be generated and presented during viral infections, where β cell destruction could be triggered. Even if the infection is cleared and regulatory mechanisms are in place, additional infections could trigger further waves of β cell destruction. This might explain why T1D has been defined as a relapsing-remitting disease, where β cells may be killed only when a certain trigger (i.e. a viral infection) is present (37). Moreover, persistent infections are likely to be problematic, as they could perpetuate inflammation, immune activation and β cell destruction. Many herpesviruses produce lifelong infections and remain in their host in a latent state. Reactivations occur upon immune dysfunction, but might also be concomitant with newer infections. Cytomegalovirus (HCMV) is one of the most prevalent viruses of this family, infecting over half of adults in the United States by the age of 40 (https://www.cdc.gov/cmv/overview.html). Several case reports have associated HCMV infection with T1D. Pak and colleagues (38) showed a strong correlation between HCMV genome and islet autoantibodies, while HCMV-positive cells were found in the islets of subjects with fulminant T1D (39). HCMV-specific CD8^+^ T cells have been found in the pancreas of T1D subjects at the onset of the disease (40). Conversely, several reports have found neither an association with the disease (41) nor even a delay in progression of T1D (42). It is therefore possible that more virus-specific cells are present in the islets during disease progression than previously expected. Other highly prevalent herpesviruses like Epstein-Barr virus (EBV), human herpesvirus 6A (HHV-6A) and 6B (HHV-6B) have also been associated with T1D. High circulating antibody titers against EBV have been detected in patients with T1D when compared to non-diabetic controls (43). Interestingly, HHV-6B glycoprotein B (gB) was more expressed in the islets and exocrine pancreas of donors with T1D, as compared to non-diabetic subjects (44). However, due to the persistent and latent nature of these viruses, a direct involvement in the pathogenesis of T1D is likely to be hard to prove.

If viral-human *zwitter* epitopes associated with multiple viruses existed, immune responses could be constantly triggered, even if they were of low magnitude, with potential implication for the etiopathogenesis of T1D. Therefore, we investigated *in silico* the theoretical existence of T1D-associated viral-human *zwitter* peptides, and their potential in triggering CD8^+^ T cell-mediated autoimmunity in T1D.

## Materials and Methods

### Peptide-HLA-I Binding Affinity Prediction and Immune Epitope Database (IEDB)

The study focused on non-spliced and *cis*-spliced 9 amino acid long (9mer) peptides and HLA-A*01:01, -A*02:01, -A*03:01, -A*11:01, -A23:01, -A*24:02, -B*07:02, -B*08:01, -B*15:01, -B*35:01, -B*39:06, -B*40:01, -B*44:02, -B44:03 complexes. This pool of HLA-I alleles covers over 90% of the Caucasian population. For each HLA-I allele, we computed a cut-off comparable to the threshold of a predicted IC_50_ ≤ 500 nM of peptide-HLA-A*02:01 complex as follows: we downloaded all 9mer peptides detected through peptide elution from HLA-I complexes, and reported in the IEDB database (45). We restricted the analysis to the HLA-I alleles specified above. For each peptide-HLA-I complex, we predicted the inhibitory constant (IC_50_) of these 9mer peptide sequences by using NetMHCpan-BA4.0 algorithm (46). IC_50_ estimates the binding affinity of HLA-I-peptide complexes. The lower the IC_50_, the higher the binding affinity between peptide and HLA-I complex. To have a similar IC_50_ cut-off among HLA-I alleles, we determined the quantile of the HLA-A*02:01 for IC_50_ = 500 nM, which resulted in 91.4%-ile of peptides present in the HLA-A*02:01-specific HLA-I immunopeptidome database of the IEDB. We then applied this quantile to the predicted IC_50_ distributions of all other peptide-HLA-I complexes (**Figure S1**), thereby identifying the IC_50_ cut-offs of each HLA-I allele, which corresponded to the peptide-HLA-A*02:01 IC_50_ = 500 nM. Values are displayed in **Table S2**.

For the identification of peptides already determined in HLA-I immunopeptidomics or analyzed (with positive outcome) for T cell recognition, we consulted the IEDB. We downloaded and selected all HLA-I-restricted peptides for which a positive T cell assay was reported (45). The latter included experiments, for example, performed through tetramer staining, IFN-γ assays with co-culture of APCs pulsed with synthetic peptide candidates and either peripheral blood mononuclear cells (PBMCs) or CD8^+^ T cell clones as well as Cr^51^ cytotoxicity. For the computation of antigenic hotspot regions see below.

### Estimation of Viral-Human *Zwitter* Peptides

Viral proteomes were obtained *via* ViralZone, and the Human proteome referred to Swiss-Prot Version 2016 excluding protein isoforms (47, 48). Only viruses with human tropism and association to T1D were included in any downstream analysis here presented (n = 8; **Table S1**). The Human proteome database contained 20,191 protein entries with a total of 11,323,862 amino acid residues.

We focused our study on 9mer peptides since they represent the majority of non-spliced and *cis*-spliced peptides in HLA-I immunopeptidomes (21, 49, 50).

We defined as viral-human *zwitter* 9mer peptide any 9mer peptide that had a sequence that could be obtained by either peptide hydrolysis or *cis*-peptide splicing, both from self-proteins and from viral proteins.

We first computed all possible non-spliced 9mer peptides from viral and human proteomes, and all normal and reverse *cis*-spliced 9mer peptides - with an intervening sequence length ≤ 25 amino acids - that could be derived from the viral and human proteomes. *Cis*-spliced peptides were computed *in silico* as previously described (49). We used an intervening sequence length restriction of 25 amino acid residues to be consistent with our previous study on HLA-I immunopeptidomes and *in cellulo* study on a tumor-associated spliced epitope (49, 51).

After, we compared all viral peptides with human peptides by aligning their sequences.

Two 9mer peptides were considered as identical, *i.e.* as viral-human *zwitter* peptides, if all of their 9 amino acid residues were exactly matching. The relative frequency of viral-human *zwitter* peptides (*Fv*) was calculated as:

$$F_{v}=100\frac{z_{v}}{p_{v}},$$

where *z_v_* is the number of all unique viral-human *zwitter* peptides of a given virus *v*; and *p_v_* is the number of all possible unique 9mer peptides derived from virus *v*. The number of viral-human *zwitter* peptides, z, was computed for the comparison of non-spliced peptides only, of *cis*-spliced peptides only, of non-spliced viral peptides compared to *cis*-spliced human peptides, and of *cis*-spliced viral peptides compared to non-spliced human peptides. Additionally, the relative frequency of all (non-spliced and *cis*-spliced) viral-human *zwitter* peptides was computed.

### Estimation of Viral-Human *Zwitter* Epitope Candidates Considering Antigenic Hotspots and the Potential Antigen Repertoire of Human mTECs and Pancreatic β Cells

To determine the potential hotspot regions among antigens that might be the origin of *zwitter* epitope candidates, we collected all peptide sequences present in IEDB’s human HLA-I immunopeptidome database and mapped them to the reference proteome database. For each amino acid in the reference proteome database, we counted how many unique peptides of IEDB’s human HLA-I immunopeptidome database contained that residue. For any given *zwitter* 9mer peptide we computed the average count over the 9 residues on its sequence, which was our hotspot score. Finally, we applied a cut-off score of 1 to define hotspot regions. Therefore, a hotspot score of 1 was computed if each residue of a given 9mer peptide was identified at least once in IEDB’s human HLA-I immunopeptidome database.

To determine the potential antigen repertoire of human medullary thymic epithelial cells (mTECs) and pancreatic β cells, we extracted gene expression values from the RNA sequencing dataset of human mTECs and pancreatic β cells, published by Gonzalez-Duque and colleagues (19), for each antigen in our study. We filtered all antigens based on their expression values, such that the expression was smaller than 0.1 RPKM in mTECs and larger than 5 RPKM in pancreatic islets.

### Predicted Protein Structures

For visualization purpose, the structure of HCMV DNA primase (*UL70*) and human IA-2 (a.k.a. PTPRN) antigens was determined using iTasser (52) with default settings without inclusion or exclusion of structural templates.

### Database Source and Data Availability

The human mTEC’s and pancreatic β cell RNA sequencing data were provided by Gonzalez-Duque et al. (19), as well as the T1D-associated antigen list (**Table S3**).

The list of *zwitter* non-spliced and *cis*-spliced 9mer peptides and related information (**Tables S4, S5**) are accessible in the repository Mendeley dataset: http://dx.doi.org/10.17632/z9g9knjxgw.1

## Results

### Estimation of Viral-Human *Zwitter* Epitope Candidates Potentially Associated to T1D

**Figure 2:**
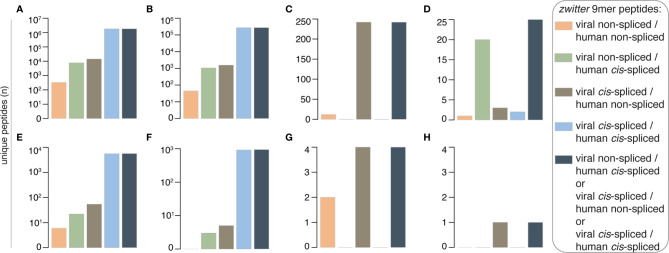
Theoretical viral-human *zwitter* 9mer peptide frequency and potential association with T1D. **(A–H)** Number of theoretical viral-human 9mer (non-spliced or *cis*-spliced) **(A)** *zwitter* peptides, **(B)** *zwitter* epitope candidates predicted to efficiently bind selected HLA-I complexes, **(C)** *zwitter* epitope candidates described in published HLA-I immunopeptidomes, **(D)** *zwitter* epitope candidates that showed a positive T cell response in published studies, **(E)** *zwitter* epitope candidates derived from T1D-associated antigens, **(F)** *zwitter* epitope candidates predicted to efficiently bind selected HLA-I complexes and derived from T1D-associated antigens, **(G)** *zwitter* epitope candidates described in published HLA-I immunopeptidomes and derived from T1D-associated antigens, **(H)** *zwitter* epitope candidates that may be derived from T1D-associated antigens and showed a positive T cell response in published studies. For the identification of epitope candidates already identified in HLA-I immunopeptidomics or analyzed (with positive outcome) for T cell recognition, we consulted the IEDB database.

For a systematic estimation of the potential number of viral-human *zwitter* epitope candidates that could (i) be presented by HLA-I complexes, (ii) be involved in an autoimmune CD8^+^ T cell response in T1D patients, and (iii) be at least in part triggered by viral infection, we started from the foundations: we computed the number of 9mer peptides that might originate from human proteome, as well as those that might originate from T1D-associated viruses (**Table S3**). We focused on 9mer peptides because this is the predominant length in HLA-I immunopeptidomes. We neglected TCR degeneracy (see Discussion), and therefore we focused only on peptides that might be derived from either human proteome and virus proteome with the exact same sequence of amino acids, here named viral-human *zwitter* peptides. With these restrictions, 332 non-spliced peptides were obtained that might be viral-human *zwitter* non-spliced 9mer peptides (**Figure 2A** and **Table S4**). Only HHV-6A and -6B, EBV and HCMV potentially carried these peptides. Among them, 45 were predicted to efficiently bind at least one of the selected HLA-I variants, which represents a large section of the Caucasian population (**Figure 2B**). Twelve viral-human *zwitter* non-spliced 9mer epitope candidates have already been eluted from HLA-I complexes and identified by mass spectrometry, and for one peptide a positive T cell assay has been described, according to the IEDB (**Figures 2C, D** **)**. Six viral-human *zwitter* non-spliced 9mer peptides could be derived from a list of T1D-associated antigens proposed by Gonzalez-Dunque et al. (19), (**Figure 2E**). None of these latter peptides were predicted to efficiently bind the selected HLA-I alleles, although two of them were identified in HLA-I immunopeptidomes, according to the IEDB (**Figures 2F, G** **)**. No viral-human *zwitter* non-spliced 9mer epitope candidates derived from T1D-associated antigens has been tested through T cell assays with a positive outcome (**Figure 2H**).

As expected, the scenario changed when we included *cis*-spliced peptides (**Table S5**). Indeed, the number of *zwitter* peptides that may be produced by *cis*-peptide splicing of either both viral and human antigens or only one or the other – herein defined as *zwitter cis*-spliced peptides - increased in each of the categories analyzed. Almost two million viral-human *zwitter cis*-spliced 9mer peptides were computed, which could be derived from the investigated viruses (**Figure 2A**), and more than 270,000 of them were predicted to efficiently bind the selected HLA-I variants (**Figure 2B**). 242 viral-human *zwitter cis*-spliced 9mer epitope candidates have already been eluted from HLA-I complexes, and identified by mass spectrometry, according to the IEDB (**Figure 2C**). However, they all belonged to the *zwitter* viral *cis*-spliced/human non-spliced peptide category; hence, they were all identified as human non-spliced peptides in human HLA-I immunopeptidomes (**Table S5**). For 25 viral-human *zwitter cis*-spliced 9mer epitope candidates, we identified studies showing a positive T cell assay (**Figure 2D**). Among them, 20 were viral non-spliced human *cis*-spliced epitope candidates, and the response has been detected against the viral non-spliced epitopes. The remaining five were viral *cis*-spliced epitopes and either human non-spliced or human *cis*-spliced epitope candidates in our database (**Table S5**). For them, the positive T cell response reported by other groups was either against the human non-spliced peptide, or a viral non-spliced peptide derived from a different viral strain than what was included in our database (**Table S1**).

**Figure 3:**
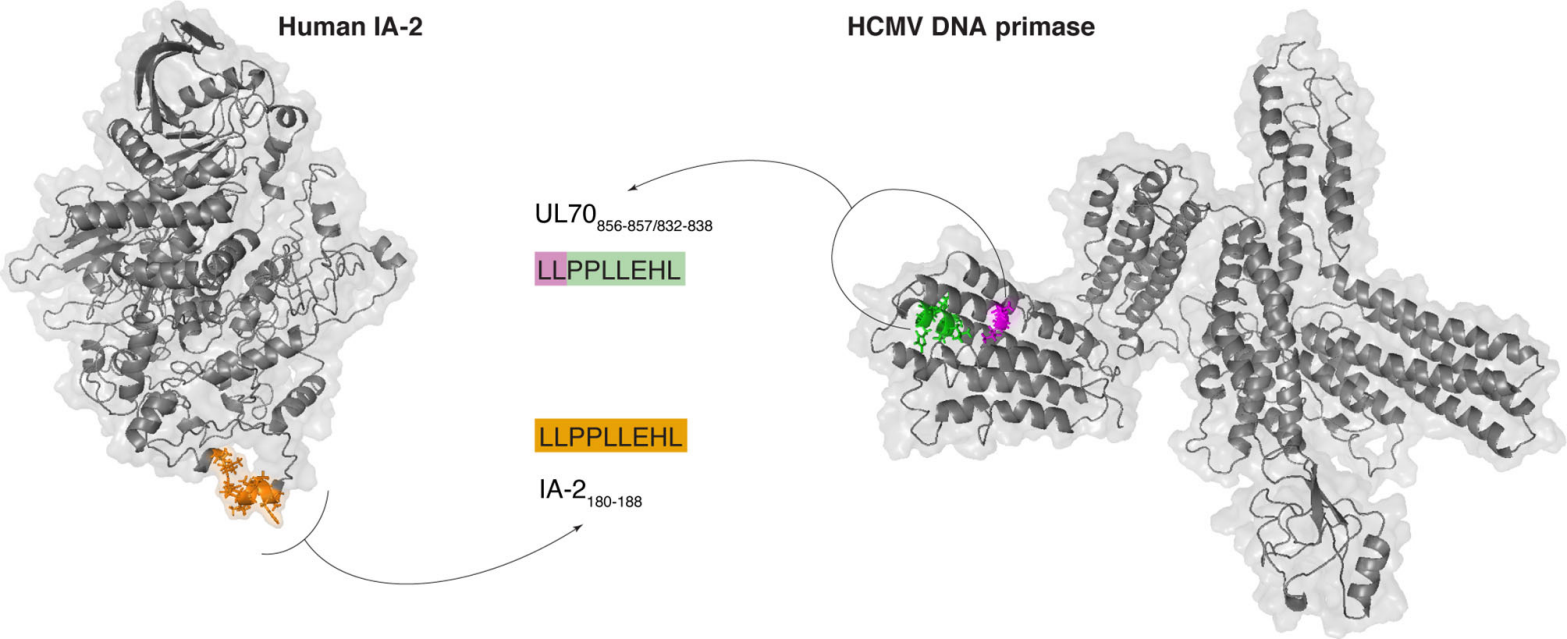
Example of potentially immunogenic *zwitter* viral *cis*-spliced/human non-spliced peptide. Predicted crystal structure of the human IA-2 (a.k.a. PTPRN) and HCMV DNA primase (*UL70*) and theoretical localization of the viral-human *zwitter* peptide candidate LLPPLLEHL. This peptide may be generated through peptide hydrolysis from human IA-2 and through peptide splicing from HCMV DNA primase. The *zwitter* non-spliced peptide candidate IA-2_180-188_ [LLPPLLEHL] is depicted in orange. For the *zwitter cis*-spliced peptide candidate UL70_856-857/832-838_ [LL][PPLLEHL], the two splice-reactants are depicted, too.

Over 5,000 viral-human *zwitter cis*-spliced 9mer peptides could be derived from the Gonzalez-Dunque’s et al. T1D-associated antigen list (**Figure 2E**). Almost a thousand of these latter peptides were also predicted to efficiently bind the selected HLA-I alleles (**Figure 2F**), and four of them were identified as non-spliced peptides in human HLA-I immunopeptidomes, according to the IEDB (**Figure 2G**). One viral-human *zwitter cis*-spliced 9mer peptide derived from T1D-associated antigens has been tested through T cell assays with a barely positive outcome (**Figure 2H**). It was the antigenic peptide [LLPPLLEHL], which may be generated through peptide hydrolysis from the human insulinoma-associated antigen 2 (IA-2; a.k.a. PTPRN) as well as, according to our computation, from the DNA primase (*UL70*) antigen of HCMV through *cis*-peptide splicing (**Figure 3**). This antigenic peptide is presented by HLA-A*02:01 complex (predicted IC_50_ = 45 nM; measured IC_50_ = 444 nM) (53, 54). In a standard IFN-γ ELISpot assay with PBMCs, only 1 out of 11 T1D HLA-A*02:01 patients showed a CD8^+^ T cell response above the cut-off, whereas no response was detected in healthy donors against this epitope (53).

### Prioritization of Viral-Human *Zwitter* Peptide Candidates Potentially Associated to T1D

Not all non-spliced and *cis*-spliced epitope candidates that might be generated are in fact produced and presented by HLA-I complexes. APP pathway has many steps that filter peptides based on their sequence and amount (5). The number of peptide molecules in HLA-I immunopeptidomes seems to depend on the amount of the antigen, its degradation rate, and the location of the peptide within antigens. The latter is well represented by “hotspot” regions in antigens, which are overrepresented by antigenic non-spliced and *cis*-spliced peptides in HLA-I immunopeptidomes (49, 55). Furthermore, RPKMs of RNA sequencing measurements, which may be an indirect indicator of protein translation, showed a certain degree of correlation with HLA-I immunopeptidomes (55). According to the current models of thymic negative selection, we could speculate that autoreactive CD8^+^ T cell clones may be more likely to recognize self-antigenic peptides that are not (or barely) presented by mTECs or thymic dendritic cells (DCs), which would reduce the risk of being eliminated during thymic negative selection. If the same antigenic peptides were highly expressed by β cells, the risk of autoreactive CD8^+^ T cell response against these cells would be higher, although several peripheral tolerance pathways are in place to repress undesired autoimmune reactions.

**Figure 4:**
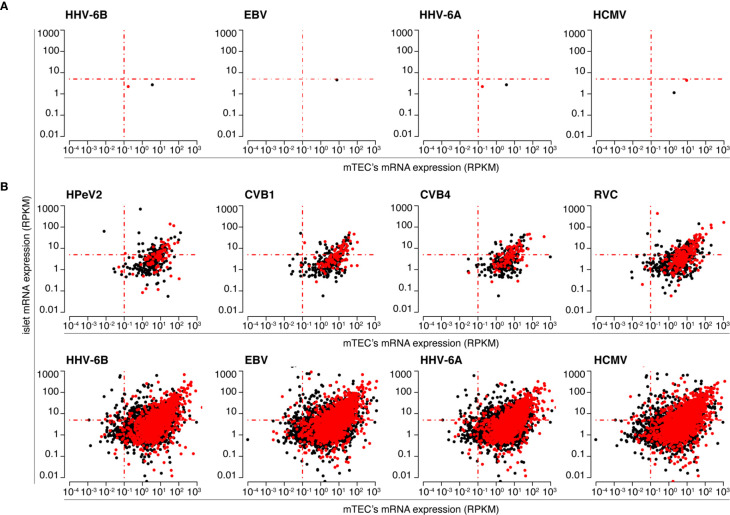
Human pancreatic islets and mTECs’ mRNA expression of antigens potentially carrying HLA-A*02:01-restricted viral-human non-spliced and *cis*-spliced *zwitter* peptide candidates. The scatter plots depict the distribution of RPKM of mRNA of human antigens, as measured by Gonzalez-Duque and colleagues (19) in human pancreatic islets and mTECs, that theoretically can carry viral-human *zwitter* **(A)** non-spliced and **(B)** *cis*-spliced epitope candidates. Scatter plots are divided based on the corresponding theoretical virus origin. In **(A)** only four out of eight viruses are shown because for four viruses no viral-human non-spliced peptide candidates with the required characteristics were identified. Black dots represent antigens carrying epitope candidates predicted to bind the HLA-A*02:01 allele. Red dots represent antigens carrying epitope candidates predicted to bind the HLA-A*02:01 allele and located in hotspots, according to the IEDB database.

With this in mind, we prioritized viral-human *zwitter* 9mer peptide candidates (predicted to bind the selected HLA-I variants) based on: (i) RNA sequencing data of human mTECs and primary pancreatic islets (for antigen selection); (ii) localization of epitope candidates within hotspot regions of antigens; (iii) antigen association with T1D. The former and the latter databases were derived from Gonzalez-Duque and colleagues (19). The localization within antigenic hotspot regions was computed based on published HLA-I immunopeptidome databases (see Material and Methods section). The distribution of RPKM of genes coding for antigens from which viral-human *zwitter* 9mer epitope candidates may be derived - as measured in mTECs and primary pancreatic islets - is reported in **Figure 4** and **Figures S2–S14**.

**Figure 5:**
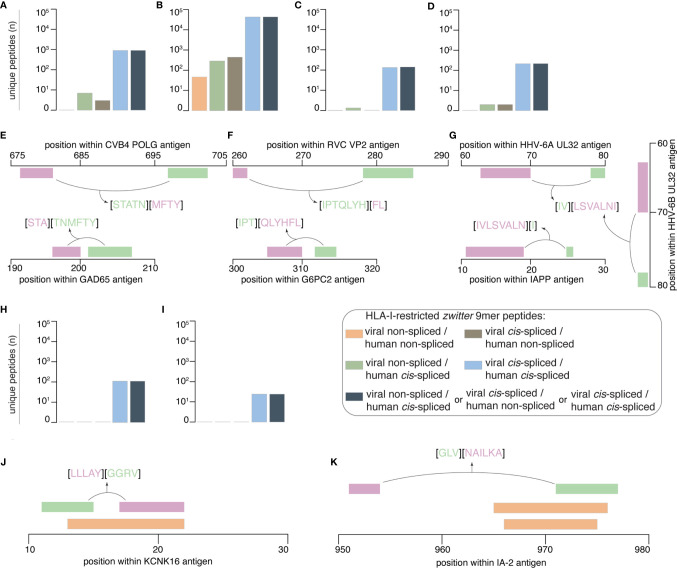
Prioritization of viral-human *zwitter* 9mer peptide candidates and examples. **(A–D)** Number of theoretical viral-human *zwitter* non-spliced or *cis*-spliced 9mer epitope candidates predicted to efficiently bind selected HLA-I variants and either **(A)** derived from antigens preferentially expressed in pancreatic islets over mTECs, or **(B)** located in hotspot regions, or **(C)** located in hotspot regions of antigens preferentially expressed in pancreatic islets over mTECs, or **(D)** derived from T1D-associated antigens preferentially expressed in pancreatic islets over mTECs. **(E–G)** Examples of *zwitter* viral-human *cis*-spliced epitope candidates derived from T1D-associated antigens preferentially expressed in pancreatic islets over mTECs: **(E)** GAD65_198-200/202-207_ [STA][TNMFTY] and CVB4-derived POLG_698-702/677-680_ [STATN][MFTY], **(F)** G6PC2_312-314/305-310_ [IPT][QLYHFL] and RVC-derived VP2_278-284/260-261_ [IPTQLYH][FL], as well as **(G)** IAPP_11-18/25_ [IVLSVALN][I], HHV-6A-derived UL32_79-80/64-70_ [IV][LSVALNI] and HHV-6B-derived UL32_79-80/64-70_ [IV][LSVALNI] *cis*-spliced peptides. **(H, I)** Number of theoretical viral-human *zwitter* non-spliced or *cis*-spliced 9mer epitope candidates derived from T1D-associated antigens, predicted to efficiently bind the selected HLA-I variants and either **(H)** located in hotspot regions, or **(I)** located in hotspot regions of antigens preferentially expressed in pancreatic islets over mTECs. **(J, K)** Examples of *zwitter* viral-human *cis*-spliced peptide candidates potentially associated to T1D and located in hotspots: **(J)** KCNK16_17-21/11-14_ [LLLAY][GGRV] and **(K)** IA-2_951-953/971-976_ [GLV][NAILKA] *cis*-spliced epitope candidates are located in an area where non-spliced antigenic peptides (orange bars) have been identified by mass spectrometry in HLA-I immunopeptidomes by others. In **(E–G**, **J, K)** bars’ color code corresponds to that used in **Figure 1A**.

When we considered a cut-off for gene expression with a RPKM larger than 5 in islets and smaller than 0.1 in mTECs, which mimicked what was proposed by Gonzalez-Duque and colleagues (19), we obtained no viral-human *zwitter* non-spliced 9mer epitope candidates predicted to efficiently bind the selected HLA-I variants (**Figure 5A**). When we considered only epitope candidates predicted to bind the HLA-I variants and located in hotspot regions, sixteen viral-human *zwitter* non-spliced 9mer peptide candidates could be identified (**Figure 5B**). Again, the outcome is very different if *cis*-spliced peptides are included. Over 900 epitope candidates that were predicted to efficiently bind the selected HLA-I alleles, and theoretically derived from antigens preferentially expressed in pancreatic islets over mTECs – i.e. with RNA sequencing RPKM larger than 5 in islets and smaller than 0.1 in mTECs – may be viral-human *zwitter cis*-spliced 9mer epitope candidates (**Figure 5A**). Over 60,000 viral-human *zwitter* 9mer epitope candidates predicted to efficiently bind the selected HLA-I alleles and located in hotspot regions may have at least one of the two paired peptides produced by peptide splicing (**Figure 5B**). Among them, over a hundred were predicted to efficiently bind the selected HLA-I alleles, derived from antigens preferentially expressed in pancreatic islets over mTECs and located in hotspot regions. None of the viral-human *zwitter* non-spliced 9mer epitope candidates had these characteristics (**Figure 5C**). When we focused our *in silico* investigation on T1D-associated antigens (**Table S3**), although no non-spliced peptides were predicted to efficiently bind the selected HLA-I alleles, and may be derived from antigens within the genes’ RPKM cut-offs, over 200 viral-human *zwitter* 9mer epitope candidates with such characteristics may have at least one of the two paired peptides produced by peptide splicing (**Figure 5D**).

From the latter, we mention the example of the *zwitter cis*-spliced epitope candidate, which may be derived from the human GAD65 antigen as [STA][TNMFTY] and from the CVB4 Genome Polyprotein POLG as [STATN][MFTY] (**Figure 5E**). This *zwitter cis*-spliced 9mer epitope candidate was predicted to bind HLA-A*01:01, -A*11:01 and -B*35:01 with IC_50_ < 100 nM (**Table S5**). In this pool of epitope candidates, we also have a peptide that may be derived from RVC, and specifically from the Inner Capsid Protein VP2 as VP2_278-284/260-261_ [IPTQLYH][FL]. The human counterpart would be the peptide [IPT][QLYHFL] derived from Glucose-6-phosphatase 2 (G6PC2_312-314/305-310_), which was predicted to bind HLA-B*07:02 and -B*35:01 with IC_50_ < 200 nM (**Table S5**, **Figure 5F**). This pool of *zwitter cis*-spliced 9mer epitope candidates also contained many peptides that may be derived from HHV-6A and -6B. In some cases, the viral *zwitter cis*-spliced peptide may be derived from both HHV-6A and -6B, as the peptide [IV][LSVALNI], *i.e.* from HHV-6A Packaging protein UL32 and HHV-6B UL32 homolog (**Figure 5G**). The human counterpart would be the *cis*-spliced peptide [IVLSVALN][I] derived from Islet amyloid polypeptide (IAPP), which is predicted to efficiently bind the HLA-A*02:01 complex (**Table S5**).

When we considered peptides derived from hotspot regions of T1D-associated antigens and predicted to efficiently bind the selected HLA-I alleles, no non-spliced and over 100 *cis*-spliced epitope candidates were identified (**Figure 5H**). Among them, twenty-five may be derived from antigens whose genes were highly expressed in pancreatic islets and marginally expressed in mTECs (**Figure 5I**). The viral epitope candidates may be derived from either EBV or HCMV antigens (**Table S5**). The two T1D-associated antigens are IA-2 and Potassium channel subfamily K member 16 (KCNK16). For the latter, we mention the example of the human KCNK16_17-21/11-14_ *cis*-spliced peptide candidate [LLLAY][GGRV], which may also be annotated as EBV Glycoprotein 42 BZLF2_21-25/34-37_. If these two *cis*-spliced epitope candidates were actually generated by proteasomes, it would be through the ligation of the same splice-reactants [LLLAY] and [GGRV]. The KCNK16_17-21/11-14_ *cis*-spliced peptide was predicted to efficiently bind HLA-A*02:01 complex (predicted IC_50_ = 93 nM; **Table S5**). It partially overlapped with the HLA-A*03:01-restricted non-spliced peptide KCNK16_13-21_ [RVLPLLLAY] (**Figure 5J**), which was identified in HLA-I immunopeptidomes of human ECN90 pancreatic β cell line upon IFN-γ stimulation (19).

The other half of the viral-human *zwitter cis*-spliced epitope candidates that are included in this final list may be derived from IA-2 protein, which was largely expressed in human pancreatic islets whereas it was barely expressed in human mTECs (**Figure 4**). Among them, we briefly describe the *zwitter* IA-2_951-953/971-976_ *cis*-spliced peptide candidate [GLV][NAILKA], which may also be annotated as EBV Major DNA-binding protein DBP_853-855/835-840_. Also, in this case, the peptide splicing reaction would be between the same two splice-reactants [GLV] and [NAILKA]. The *zwitter cis*-spliced peptide IA-2_951-953/971-976_ was predicted to efficiently bind HLA-A*02:01 complex (**Table S5**). The C-terminal splice-reactant of this *cis*-spliced peptide may be derived from an IA-2 area where the non-spliced epitope candidates IA-2_966-974_ [VAEEVNAIL] and IA-2_965-975_ [AVAEEVNAILK] were also found (**Figure 5K**). The latter non-spliced epitope candidates have been predicted to bind other HLA-I alleles, and were identified in HLA-I immunopeptidomes by mass spectrometry (19, 56).

IA-2 and KCNK16 antigens are not overrepresented in the IEDB’s HLA-I immunopeptidome database (data not shown), therefore their predominance in this latter group of optimal viral-human *zwitter cis*-spliced 9mer epitope candidates may be due to a partial sequence homology between their sequence and the viral antigen sequences. This is true for IA-2 antigen, which has one of the largest pools of theoretical viral-human *zwitter cis*-spliced 9mer peptide candidates among the T1D-associated antigens (**Table S5**).

## Discussion

Our study is the first attempt to evaluate the potential role of antigenic *cis*-spliced peptides in a CD8^+^ T cell-mediated autoimmune response triggered by viral infections. Due to the theoretically extremely large pool of *cis*-spliced peptide sequences, and the limited knowledge of proteasome-catalyzed peptide hydrolysis and peptide splicing dynamics (6, 22, 23, 57–59), any *in silico* analysis of *zwitter cis*-spliced epitope candidates faces further hurdles and a higher degree of complexity when compared to analysis of canonical peptides. Therefore, in this study we neglected multiple layers of complexity of the CD8^+^ T cell response and HLA-I-restricted APP pathway, and focused only on *zwitter* 9mer peptides that share a complete homology between viral- and human-derived peptide candidates. This analysis provided a first estimation of the scale of the pool of viral-human *zwitter cis*-spliced epitope candidates potentially associated with T1D.

Where we disregarded APP pathway and antigen expression, this theoretical pool varied from a few hundred non-spliced peptides to millions of *cis*-spliced peptides. When we considered that, in the context of the CD8^+^ T cell cytotoxicity against pancreatic β cells, immunogenic epitopes are supposed to efficiently bind HLA-I clefts and to be derived from antigens (preferentially from antigen hotspots) that are expressed in pancreatic β cells and, ideally, barely expressed in mTECs, this initial figure seems to decrease. No viral-human *zwitter* 9mer non-spliced epitope candidates, and a hundred *cis*-spliced epitope candidates were left. On the one hand, this figure can further shrink if we considered that not all possible non-spliced and *cis*-spliced epitope candidates are actually produced by proteasomes. Based on *in vitro* digestion experiments with synthetic polypeptides and purified proteasomes, measured by mass spectrometry, we estimated that around one fourth of non-spliced and less than 0.4% of *cis*-spliced peptides that might have been produced were in fact produced by proteasomes in those conditions, and many of them in such a small amount that make them most likely immunologically irrelevant (22, 27). On the other hand, CD8^+^ TCRαβ are prone to a certain degree of degeneracy of their specificity. This might lead to cross-recognition of multiple antigenic peptides, thereby increasing the immunological overlap between self and non-self antigens. The immunological relevance of CD8^+^ TCRαβ cross-reactivity is still a matter of debate (60–63), although seminal studies on potential cross-reactivity of T1D-relevant CD8^+^ T cell clones for T1D-associated human antigens and pathogens have already been published (28, 64, 65). In future analyses of *cis*-spliced epitope candidates, including TCR degeneracy would represent a computational challenge, although it might significantly increase the number of viral-human *zwitter* epitope candidates potentially associated to T1D. With today’s limited knowledge of TCR degeneracy, we can also speculate that its introduction in the analysis would also increase the number of false viral-human *zwitter* epitope candidates. Additionally, future experimental analyses should consider the islet microenvironment, where proinflammatory molecules could promote immune activation and antigen presentation to APCs, further refining the pool of epitope candidates that could effectively be presented.

Regarding the *zwitter* epitope candidates derived from T1D-associated viruses and pancreatic β cell antigens described in this study, we found interesting examples potentially derived from CVB4, HHV-6A and -6B as well as RVC. Of course, the *in silico* identification of *zwitter* epitope candidates derived from T1D-associated viruses and pancreatic β cell antigens, even if confirmed *in vitro* and *in cellulo*, would not represent the only key to understanding T1D pathogenesis. It is likely that the strong genetic component of this disease plays a crucial role. We think, however, that in some T1D patients, an autoimmune response could be triggered by viral infection, which in turn might target viral-human *zwitter cis*-spliced epitopes. Although the exact mechanisms by which this occurs are currently unknown, it is possible that type I interferons, secreted in response to viral infections, play an important role. Exposure of human β cells to IFN-α leads to changes in chromatin accessibility, mRNA and protein expression, and the subsequent activation of pathways involved in protein modification, degradation and ER stress (66). IFN-α is capable of shaping the islet microenvironment by inducing the upregulation of several RNA-binding proteins with direct effects in immune cells and the potential to induce extensive changes in alternative splicing, activation and differentiation (66). In addition, the hyper-expression of HLA-I and other anti-viral response markers is associated with islet immune infiltration, which suggests that inflammatory and anti-viral responses play a crucial role in creating an islet microenvironment that potentially attracts APCs and favors antigen presentation (67). The mechanisms leading to epitope formation and presentation, as well as its possible outcomes in terms of T cell activation and cytotoxicity remain elusive, and thus will need to be elucidated in future.

## Data Availability Statement

The datasets presented in this study can be found in online repositories. The names of the repository/repositories and accession number(s) can be found in the article/**Supplementary Material**.

## Author Contributions

JL and MM designed the study. JL and AM carried out the data analysis and MM the data mining. MM and TR-C critically revised the immunological implication of the analysis. JL, TR-C, and MM wrote the manuscript. All authors contributed to the article and approved the submitted version.

## Funding

The study was in part supported by: (i) MPI-BPC collaboration agreement 2020, Cancer Research UK [C67500; A29686] and National Institute for Health Research (NIHR) Biomedical Research Centre based at Guy’s and St Thomas’ NHS Foundation Trust and King’s College London and/or the NIHR Clinical Research Facility to MM; (ii) Helmholtz Zentrum Munich junior group funding to TR-C; (iii) European Research Council (ERC) under the European Union’s Horizon 2020 research and innovation programme (grant agreement No 945528) to JL.

## Conflict of Interest

The authors declare that the research was conducted in the absence of any commercial or financial relationships that could be constructed as a potential conflict of interest.
