# Supplementary material for: Potential Mimicry of Viral and Pancreatic β Cell Antigens Through Non-Spliced and cis-Spliced Zwitter Epitope Candidates in Type 1 Diabetes
Source: Front Immunol. 2021 Apr 15;12:656451. doi: 10.3389/fimmu.2021.656451 (PMC8082463; doi:10.3389/fimmu.2021.656451)
Supplement: Supplementary file 1 [file DataSheet_1.pdf]

Supplementary information

Potential mimicry of viral and pancreatic β cell antigens through non-spliced and *cis*-spliced *zwitter* epitope candidates in Type 1 Diabetes

$ Correspondence to: michele.mishto@kcl.ac.uk, jliepe@mpibpc.mpg.de.

Table S1	List of virus strains included in the study
Table S2	Peptide-HLA-I binding affinity, reported as IC_{50} and used as cut-offs
Table S3	Gonzalez-Duque's T1D-associated antigen list
Table S4	List of viral-human *zwitter* non-spliced peptide candidates
Table S5	List of viral-human *zwitter cis*-spliced peptide candidates
Figure S1	Peptide-HLA-I binding affinity distribution of the 14 HLA-I alleles enrolled in the study
Figure S2	Human pancreatic islets and mTECs' mRNA expression of antigens potentially carrying HLA-A*01:01-restricted viral-human non-spliced and *cis*-spliced *zwitter* peptide candidates
Figure S3	Human pancreatic islets and mTECs' mRNA expression of antigens potentially carrying HLA-A*03:01-restricted viral-human non-spliced and *cis*-spliced *zwitter* peptide candidates
Figure S4	Human pancreatic islets and mTECs' mRNA expression of antigens potentially carrying HLA-A*11:01-restricted viral-human non-spliced and *cis*-spliced *zwitter* peptide candidates
Figure S5	Human pancreatic islets and mTECs' mRNA expression of antigens potentially carrying HLA-A*23:01-restricted viral-human non-spliced and *cis*-spliced *zwitter* peptide candidates
Figure S6	Human pancreatic islets and mTECs' mRNA expression of antigens potentially carrying HLA-A*24:02-restricted viral-human non-spliced and *cis*-spliced *zwitter* peptide candidates
Figure S7	Human pancreatic islets and mTECs' mRNA expression of antigens potentially carrying HLA-B*07:02-restricted viral-human non-spliced and *cis*-spliced *zwitter* peptide candidates
Figure S8	Human pancreatic islets and mTECs' mRNA expression of antigens potentially carrying HLA-B*08:01-restricted viral-human non-spliced and *cis*-spliced *zwitter* peptide candidates
Figure S9	Human pancreatic islets and mTECs' mRNA expression of antigens potentially carrying HLA-B*15:01-restricted viral-human non-spliced and *cis*-spliced *zwitter* peptide candidates
Figure S10	Human pancreatic islets and mTECs' mRNA expression of antigens potentially carrying HLA-B*35:01-restricted viral-human non-spliced and *cis*-spliced *zwitter* peptide candidates
Figure S11	Human pancreatic islets and mTECs' mRNA expression of antigens potentially carrying HLA-B*39:06-restricted viral-human non-spliced and *cis*-spliced *zwitter* peptide candidates

Figure S12	Human pancreatic islets and mTECs' mRNA expression of antigens potentially carrying HLA-B*40:01-restricted viral-human non-spliced and *cis*-spliced *zwitter* peptide candidates
Figure S13	Human pancreatic islets and mTECs' mRNA expression of antigens potentially carrying HLA-B*44:02-restricted viral-human non-spliced and *cis*-spliced *zwitter* peptide candidates
Figure S14	Human pancreatic islets and mTECs' mRNA expression of antigens potentially carrying HLA-B44:03-restricted viral-human non-spliced and *cis*-spliced *zwitter* peptide candidates

Virus	Acronym	Strain
Coxsackievirus B1	CVB1	Japan
Coxsackievirus B4	CVB4	E2
Epstein-Barr virus	EBV	B95 8 HHV 4
Human cytomegalovirus	HCMV	AD169
Human herpesvirus 6A	HHV-6A	6A
Human herpesvirus 6B	HHV-6B	6B
Human parechovirus 2	HPeV2	Williamson
Rotavirus C	RVC	Isolate RVC

Table S1. List of virus strains included in the study. Viruses included in this study are listed with their respective strains and acronyms.

HLA complex	IC50 cut-off (nM)
HLA-A*02:01	500.0
HLA-A*01:01	1486.5
HLA-A*03:01	482.3
HLA-A*11:01	132.2
HLA-A*23:01	263.7
HLA-A*24:02	519.9
HLA-B*07:02	239.3
HLA-B*08:01	1687.7
HLA-B*15:01	357.7
HLA-B*35:01	153.9
HLA-B*39:06	5574.7
HLA-B*40:01	171.0
HLA-B*44:02	550.0
HLA-B*44:03	650.2

Table S2. Peptide-HLA-I binding affinity, reported as IC_{50} and used as cut-offs. These cut-offs corresponded to 91.4%-ile of peptides present in the HLA-I immunopeptidome databases of IEDB database [1].

Table S3. Gonzalez-Duque's T1D-associated antigen list. This list of T1D-associated antigens was published by Gonzalez-Duque and colleagues [2] as Figure 2B in the cognate paper. The file is accessible as Supplementary material in Frontiers in Immunology.

Table S4. List of viral-human *zwitter* non-spliced peptide candidates. This list includes viral-human *zwitter* 9mer viral non-spliced peptides. The file is accessible in the repository Mendeley dataset: http://dx.doi.org/10.17632/z9g9knjxgw.1

Table S5. List of viral-human *zwitter cis*-spliced peptide candidates. This list includes theoretical *zwitter* 9mer viral non-spliced / human *cis*-spliced, viral *cis*-spliced / human non-spliced and viral *cis*-spliced / human *cis*-spliced peptides. Unless differently stated, they are all grouped under the definition of viral-human *zwitter cis*-spliced peptide candidates in this manuscript. The file is accessible in the repository Mendeley dataset: http://dx.doi.org/10.17632/z9g9knjxgw.1

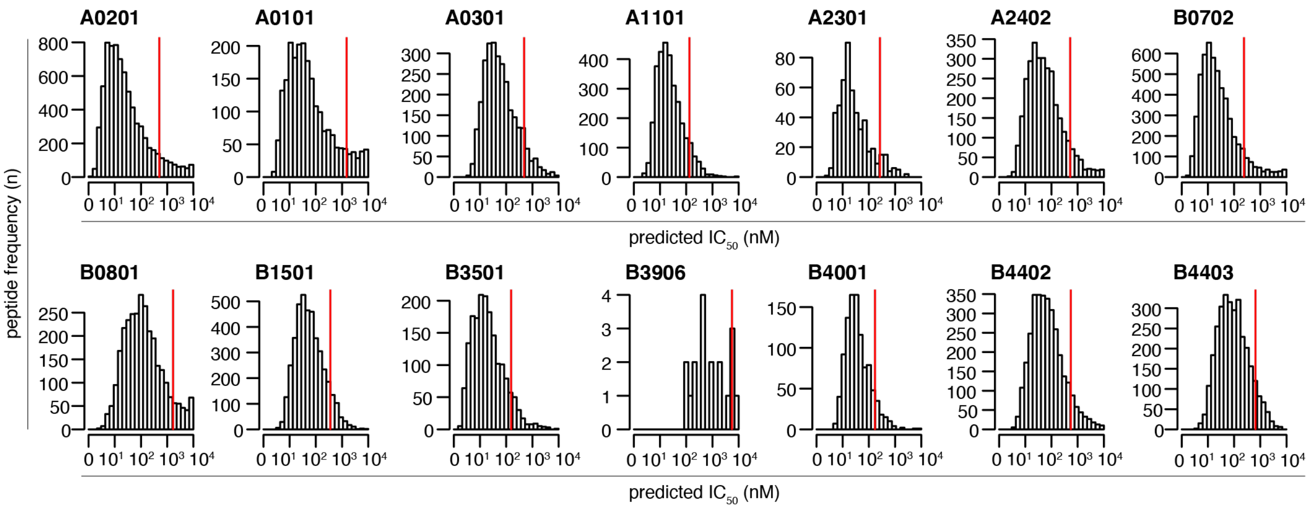

Figure S1. Peptide-HLA-I binding affinity distribution of the 14 HLA-I alleles enrolled in the study. The distribution of the binding affinity parameter IC_{50} among the HLA-I immunopeptidomes specific for the HLA-A*01:01, -A*02:01, -A*03:01, -A*11:01, -A23:01, -A*24:02, -B*07:02, -B*08:01, -B*15:01, -B*35:01, -B*39:06, -B*40:01, -B*44:02, -B44:03 alleles as reported in IEDB database. The IC_{50} was predicted by using NetMHCpan-BA4.0 algorithm [3]. Red lines represent the IC_{50} cut-off reported in **Table S2**.

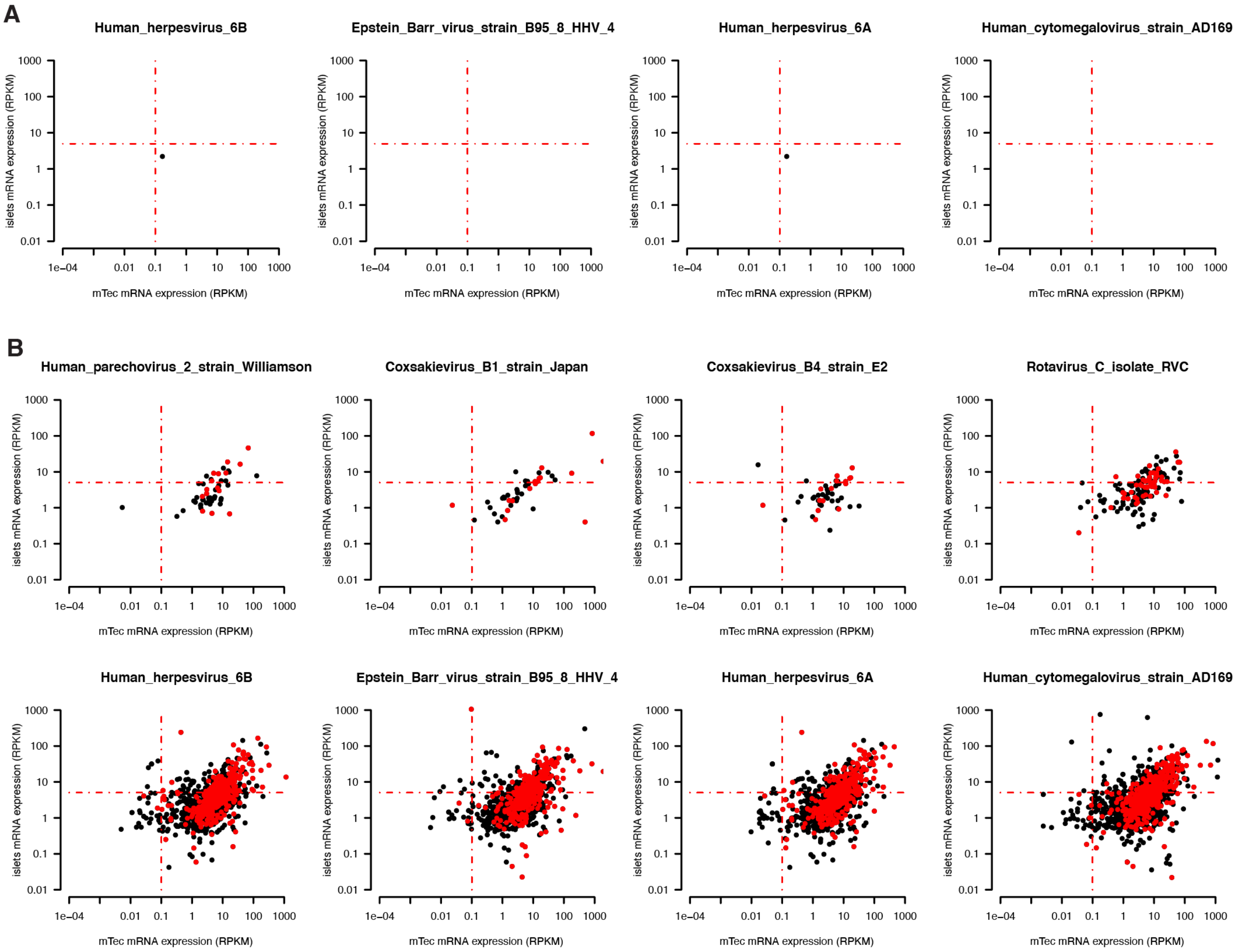

Figure S2. Human pancreatic islets and mTECs' mRNA expression of antigens potentially carrying HLA-A*01:01-restricted viral-human non-spliced and *cis*-spliced *zwitter* peptide candidates. The scatter plots depict the distribution of RPKM of mRNA of human antigens, as measured by Gonzalez-Duque and colleagues [2] in human pancreatic islets and mTECs, that theoretically can carry (**A**) non-spliced and (**B**) *cis*-spliced viral-human *zwitter* epitope candidates. Scatter plots are divided based on the corresponding theoretical virus origin. In (**A**) only four out of eight viruses are shown because for four viruses no viral-human non-spliced peptide candidates with the required characteristics were estimated. Black dots represent antigens carrying epitope candidates predicted to bind the HLA-A*01:01 allele. Red dots represent antigens carrying epitope candidates predicted to bind the HLA-A*01:01 allele and located in hotspots, according to IEDB database.

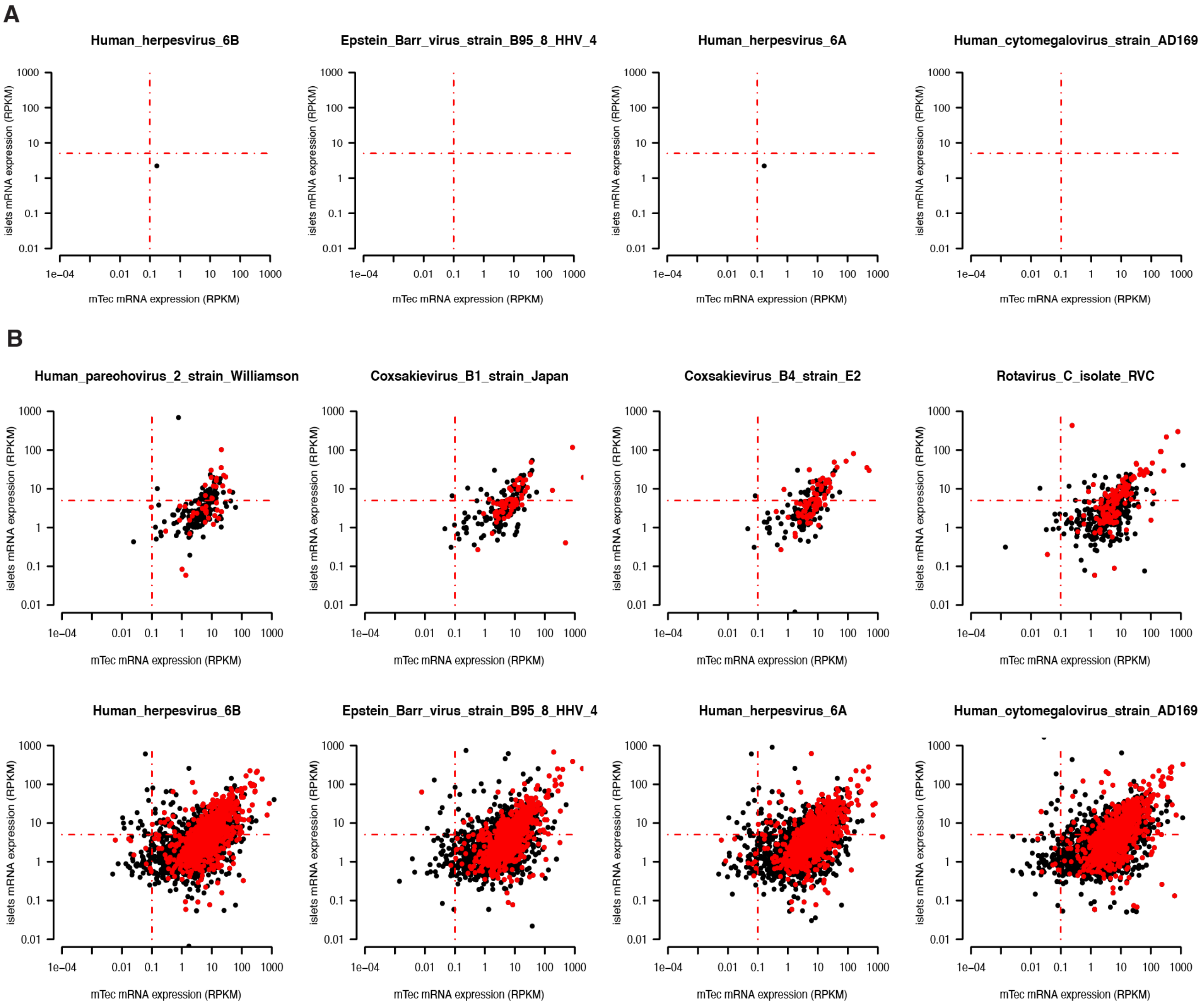

Figure S3. Human pancreatic islets and mTECs' mRNA expression of antigens potentially carrying HLA-A*03:01-restricted viral-human non-spliced and *cis*-spliced *zwitter* peptide candidates. The scatter plots depict the distribution of RPKM of mRNA of human antigens, as measured by Gonzalez-Duque and colleagues [2] in human pancreatic islets and mTECs, that theoretically can carry (**A**) non-spliced and (**B**) *cis*-spliced viral-human *zwitter* epitope candidates. Scatter plots are divided based on the corresponding theoretical virus origin. In (**A**) only four out of eight viruses are shown because for four viruses no viral-human non-spliced peptide candidates with the required characteristics were estimated. Black dots represent antigens carrying epitope candidates predicted to bind the HLA-A*03:01 allele. Red dots represent antigens carrying epitope candidates predicted to bind the HLA-A*03:01 allele and located in hotspots, according to IEDB database.

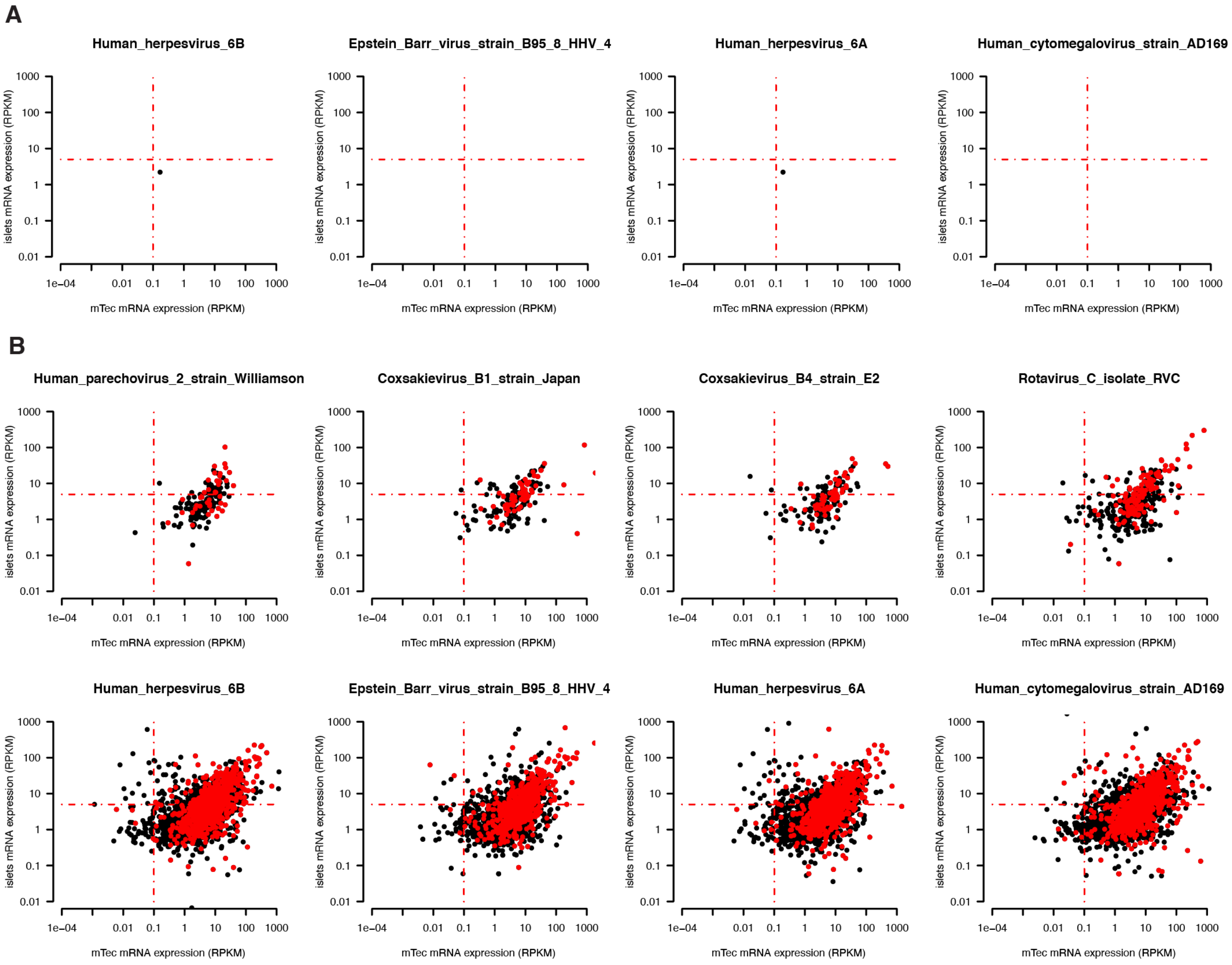

Figure S4. Human pancreatic islets and mTECs' mRNA expression of antigens potentially carrying HLA-A*11:01-restricted viral-human non-spliced and *cis*-spliced *zwitter* peptide candidates. The scatter plots depict the distribution of RPKM of mRNA of human antigens, as measured by Gonzalez-Duque and colleagues [2] in human pancreatic islets and mTECs, that theoretically can carry (**A**) non-spliced and (**B**) *cis*-spliced viral-human *zwitter* epitope candidates. Scatter plots are divided based on the corresponding theoretical virus origin. In (**A**) only four out of eight viruses are shown because for four viruses no viral-human non-spliced peptide candidates with the required characteristics were estimated. Black dots represent antigens carrying epitope candidates predicted to bind the HLA-A*11:01 allele. Red dots represent antigens carrying epitope candidates predicted to bind the HLA-A*11:01 allele and located in hotspots, according to IEDB database.

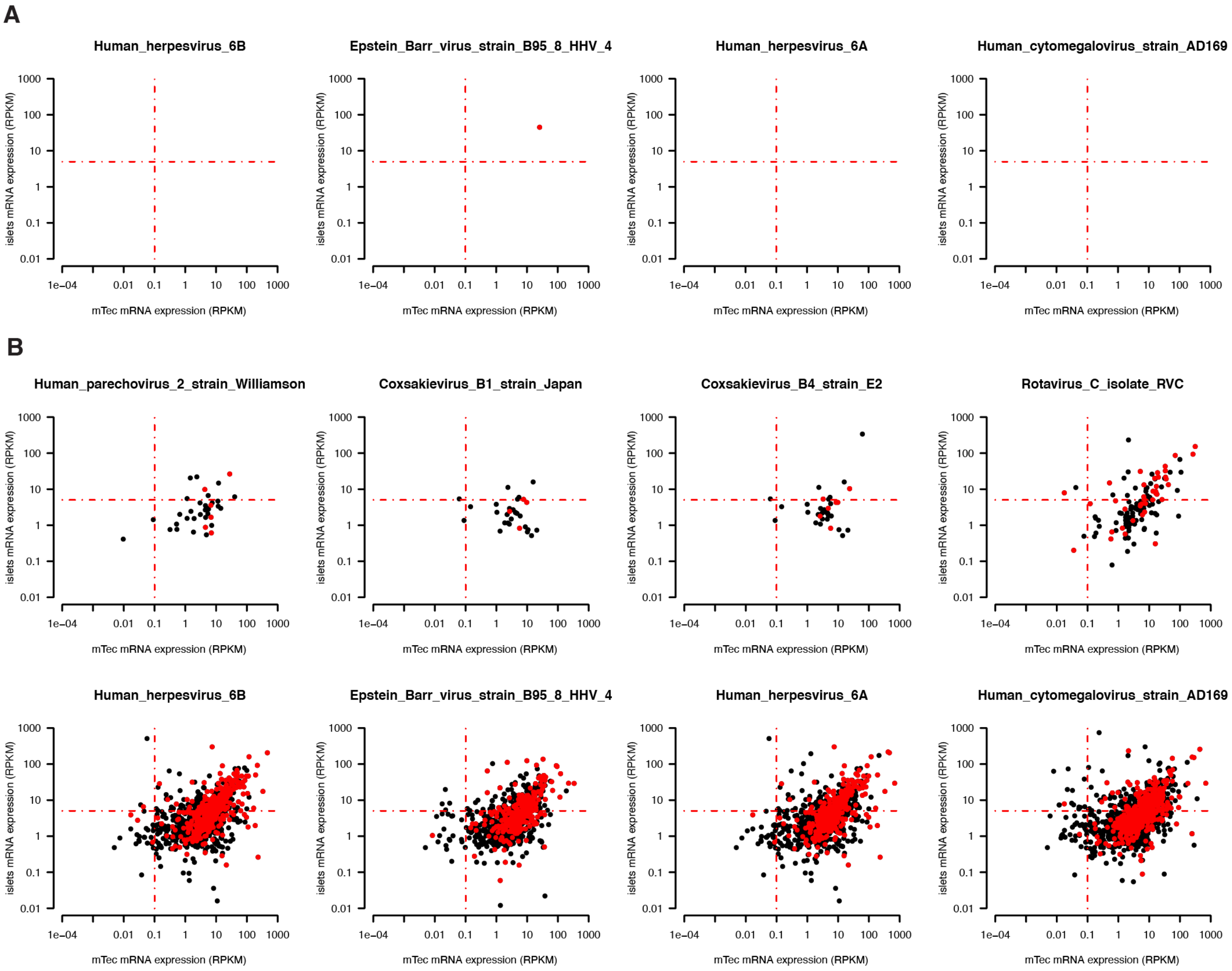

Figure S5. Human pancreatic islets and mTECs' mRNA expression of antigens potentially carrying HLA-A*23:01-restricted viral-human non-spliced and *cis*-spliced *zwitter* peptide candidates. The scatter plots depict the distribution of RPKM of mRNA of human antigens, as measured by Gonzalez-Duque and colleagues [2] in human pancreatic islets and mTECs, that theoretically can carry (**A**) non-spliced and (**B**) *cis*-spliced viral-human *zwitter* epitope candidates. Scatter plots are divided based on the corresponding theoretical virus origin. In (**A**) only four out of eight viruses are shown because for four viruses no viral-human non-spliced peptide candidates with the required characteristics were estimated. Black dots represent antigens carrying epitope candidates predicted to bind the HLA-A*23:01 allele. Red dots represent antigens carrying epitope candidates predicted to bind the HLA-A*23:01 allele and located in hotspots, according to IEDB database.

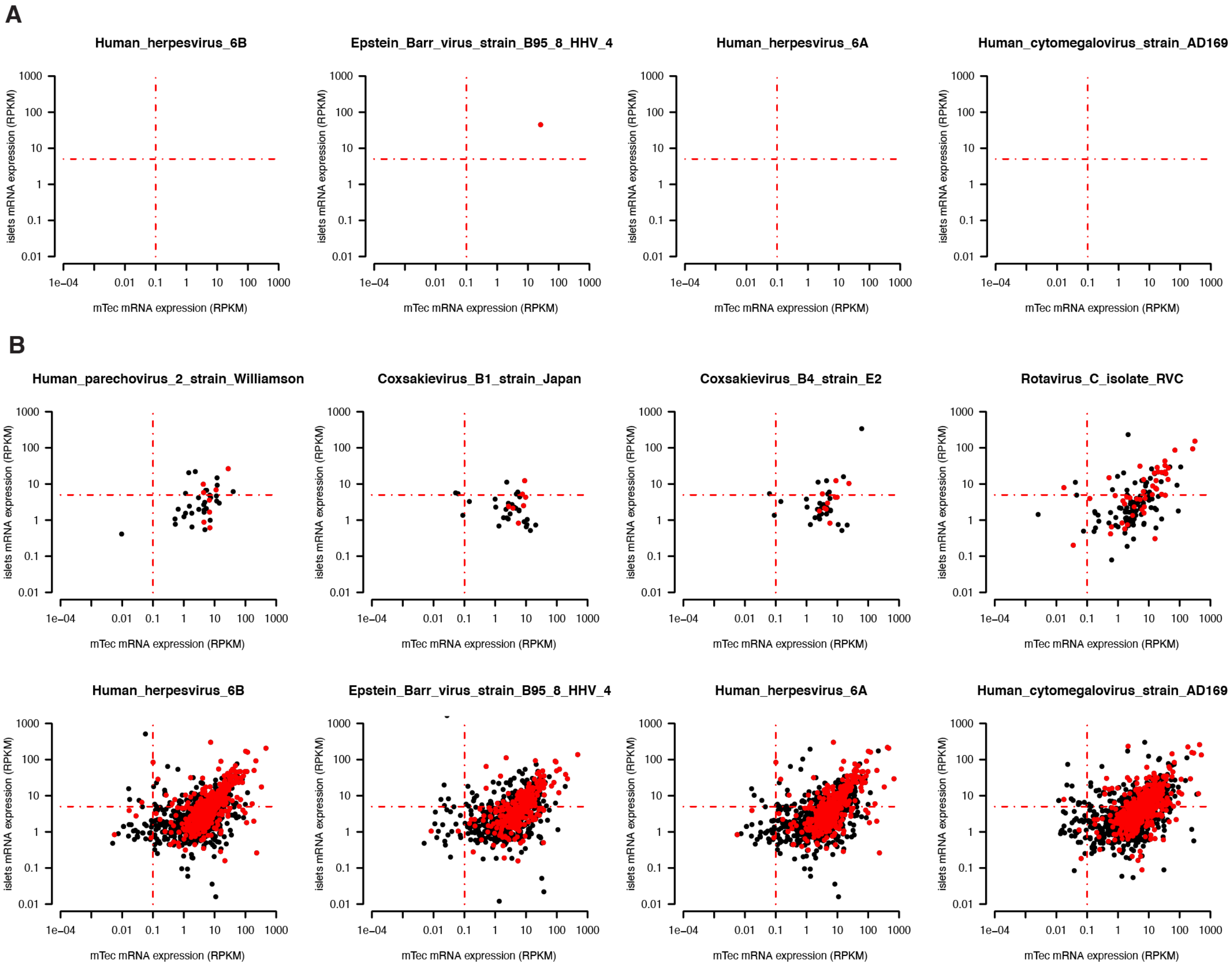

Figure S6. Human pancreatic islets and mTECs' mRNA expression of antigens potentially carrying HLA-A*24:02-restricted viral-human non-spliced and *cis*-spliced *zwitter* peptide candidates. The scatter plots depict the distribution of RPKM of mRNA of human antigens, as measured by Gonzalez-Duque and colleagues [2] in human pancreatic islets and mTECs, that theoretically can carry (**A**) non-spliced and (**B**) *cis*-spliced viral-human *zwitter* epitope candidates. Scatter plots are divided based on the corresponding theoretical virus origin. In (**A**) only four out of eight viruses are shown because for four viruses no viral-human non-spliced peptide candidates with the required characteristics were estimated. Black dots represent antigens carrying epitope candidates predicted to bind the HLA-A*24:02 allele. Red dots represent antigens carrying epitope candidates predicted to bind the HLA-A*24:02 allele and located in hotspots, according to IEDB database.

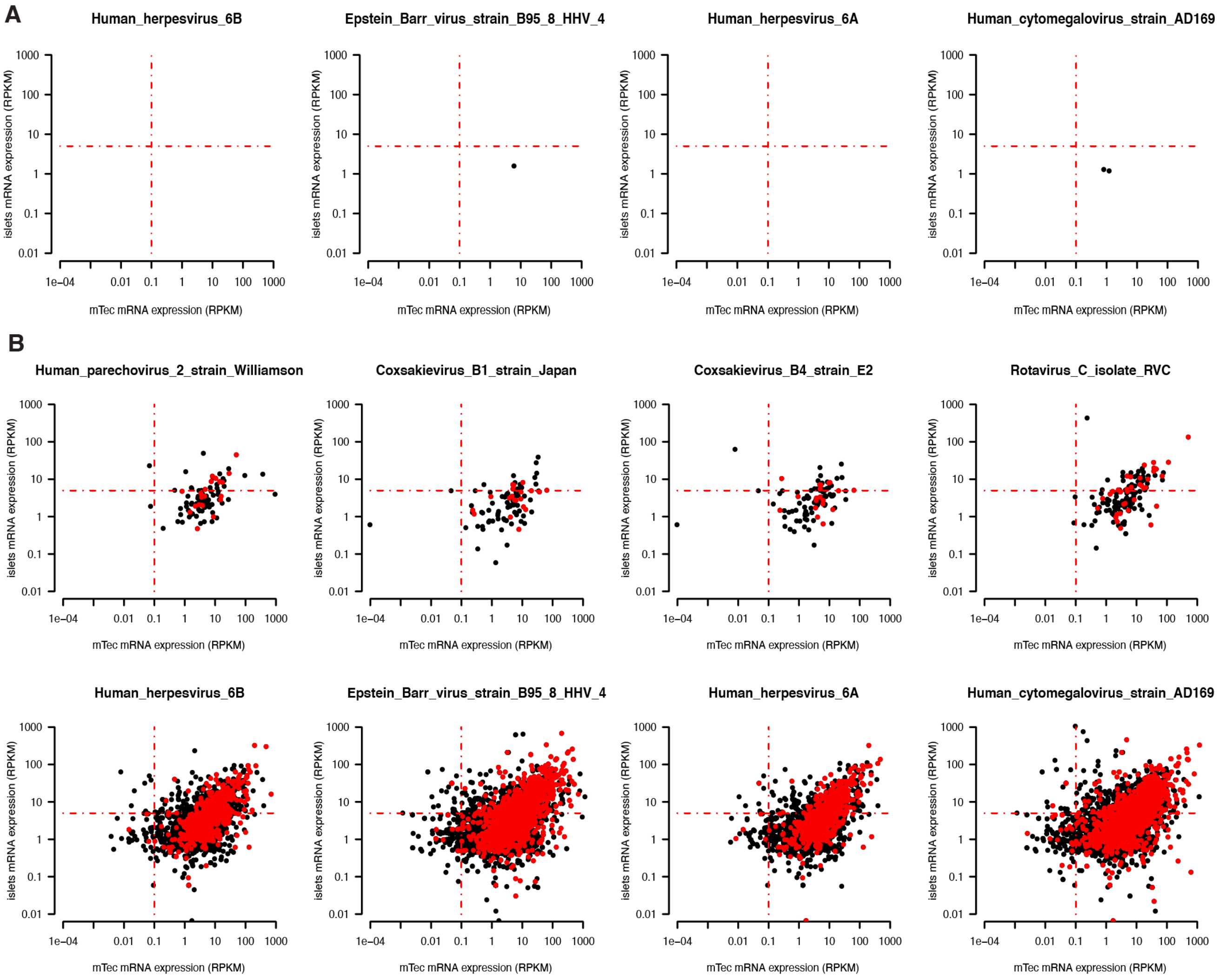

Figure S7. Human pancreatic islets and mTECs' mRNA expression of antigens potentially carrying HLA-B*07:02-restricted viral-human non-spliced and *cis*-spliced *zwitter* peptide candidates. The scatter plots depict the distribution of RPKM of mRNA of human antigens, as measured by Gonzalez-Duque and colleagues [2] in human pancreatic islets and mTECs, that theoretically can carry (**A**) non-spliced and (**B**) *cis*-spliced viral-human *zwitter* epitope candidates. Scatter plots are divided based on the corresponding theoretical virus origin. In (**A**) only four out of eight viruses are shown because for four viruses no viral-human non-spliced peptide candidates with the required characteristics were estimated. Black dots represent antigens carrying epitope candidates predicted to bind the HLA-B*07:02 allele. Red dots represent antigens carrying epitope candidates predicted to bind the HLA-B*07:02 allele and located in hotspots, according to IEDB database.

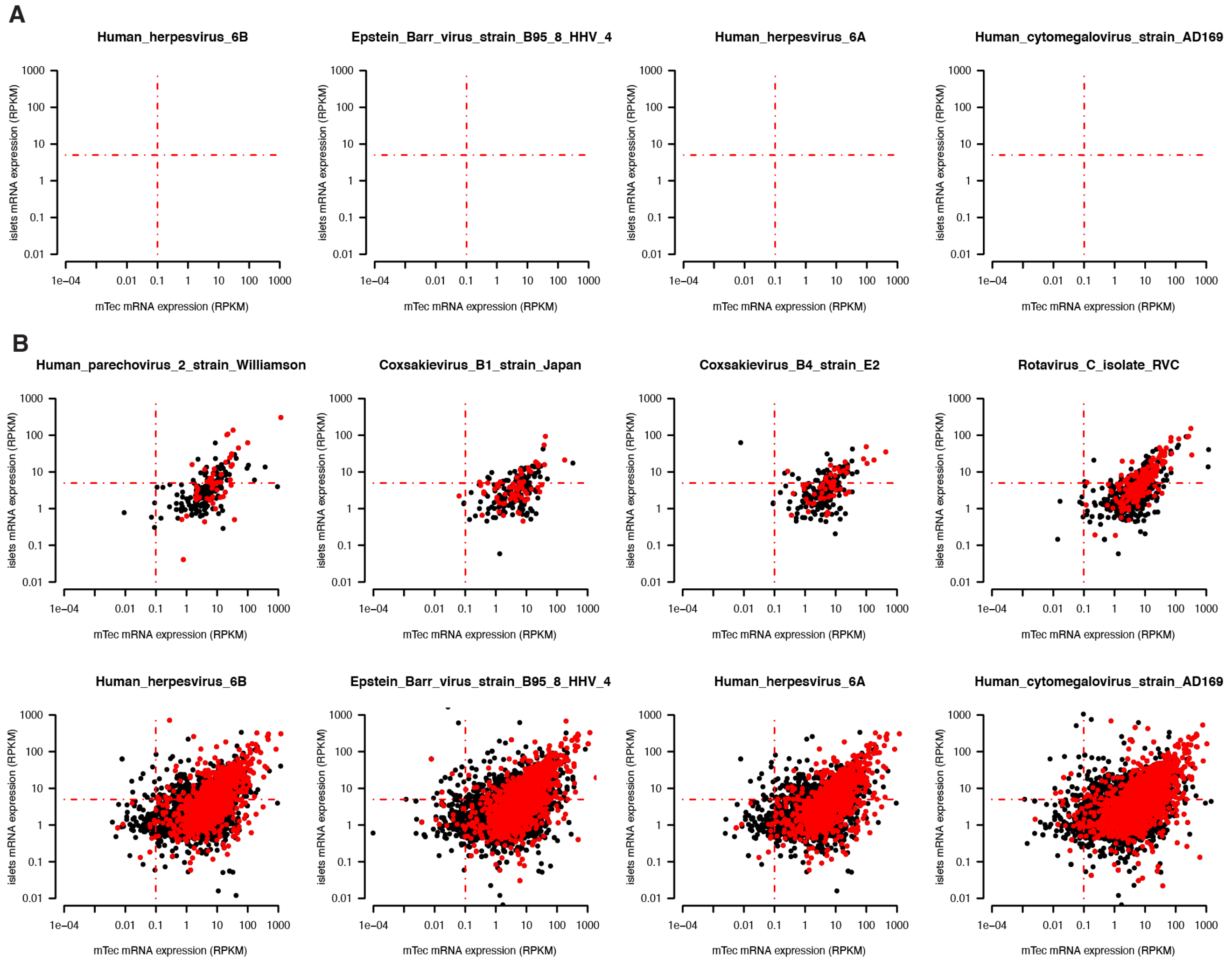

Figure S8. Human pancreatic islets and mTECs' mRNA expression of antigens potentially carrying HLA-B*08:01-restricted viral-human non-spliced and *cis*-spliced *zwitter* peptide candidates. The scatter plots depict the distribution of RPKM of mRNA of human antigens, as measured by Gonzalez-Duque and colleagues [2] in human pancreatic islets and mTECs, that theoretically can carry (**A**) non-spliced and (**B**) *cis*-spliced viral-human *zwitter* epitope candidates. Scatter plots are divided based on the corresponding theoretical virus origin. In (**A**) only four out of eight viruses are shown because for four viruses no viral-human non-spliced peptide candidates with the required characteristics were estimated. Black dots represent antigens carrying epitope candidates predicted to bind the HLA-B*08:01 allele. Red dots represent antigens carrying epitope candidates predicted to bind the HLA-B*08:01 allele and located in hotspots, according to IEDB database.

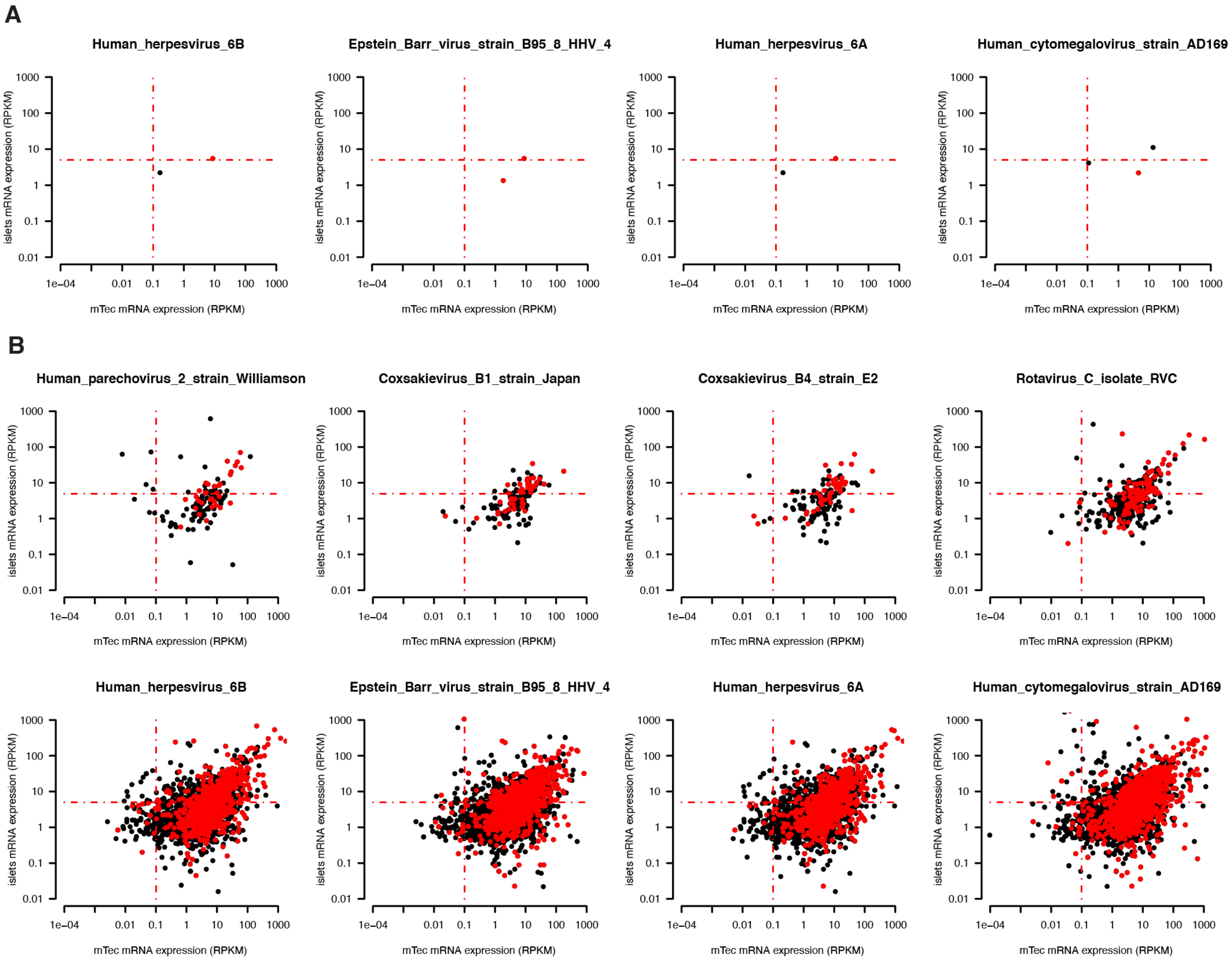

Figure S9. Human pancreatic islets and mTECs' mRNA expression of antigens potentially carrying HLA-B*15:01-restricted viral-human non-spliced and *cis*-spliced *zwitter* peptide candidates. The scatter plots depict the distribution of RPKM of mRNA of human antigens, as measured by Gonzalez-Duque and colleagues [2] in human pancreatic islets and mTECs, that theoretically can carry (**A**) non-spliced and (**B**) *cis*-spliced viral-human *zwitter* epitope candidates. Scatter plots are divided based on the corresponding theoretical virus origin. In (**A**) only four out of eight viruses are shown because for four viruses no viral-human non-spliced peptide candidates with the required characteristics were estimated. Black dots represent antigens carrying epitope candidates predicted to bind the HLA-B*15:01 allele. Red dots represent antigens carrying epitope candidates predicted to bind the HLA-B*15:01 allele and located in hotspots, according to IEDB database.

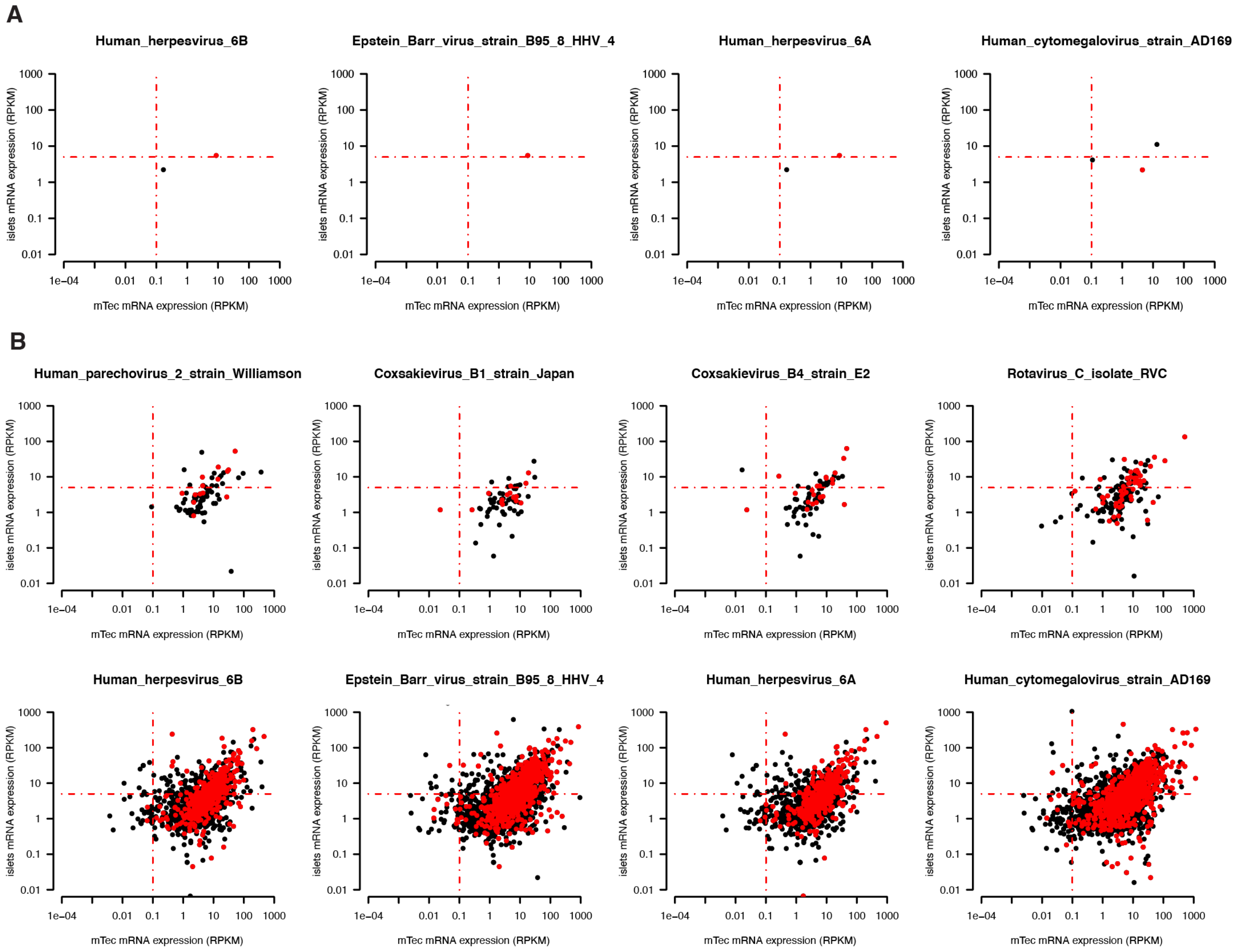

Figure S10. Human pancreatic islets and mTECs' mRNA expression of antigens potentially carrying HLA-B*35:01-restricted viral-human non-spliced and *cis*-spliced *zwitter* peptide candidates. The scatter plots depict the distribution of RPKM of mRNA of human antigens, as measured by Gonzalez-Duque and colleagues [2] in human pancreatic islets and mTECs, that theoretically can carry (**A**) non-spliced and (**B**) *cis*-spliced viral-human *zwitter* epitope candidates. Scatter plots are divided based on the corresponding theoretical virus origin. In (**A**) only four out of eight viruses are shown because for four viruses no viral-human non-spliced peptide candidates with the required characteristics were estimated. Black dots represent antigens carrying epitope candidates predicted to bind the HLA-B*35:01 allele. Red dots represent antigens carrying epitope candidates predicted to bind the HLA-B*35:01 allele and located in hotspots, according to IEDB database.

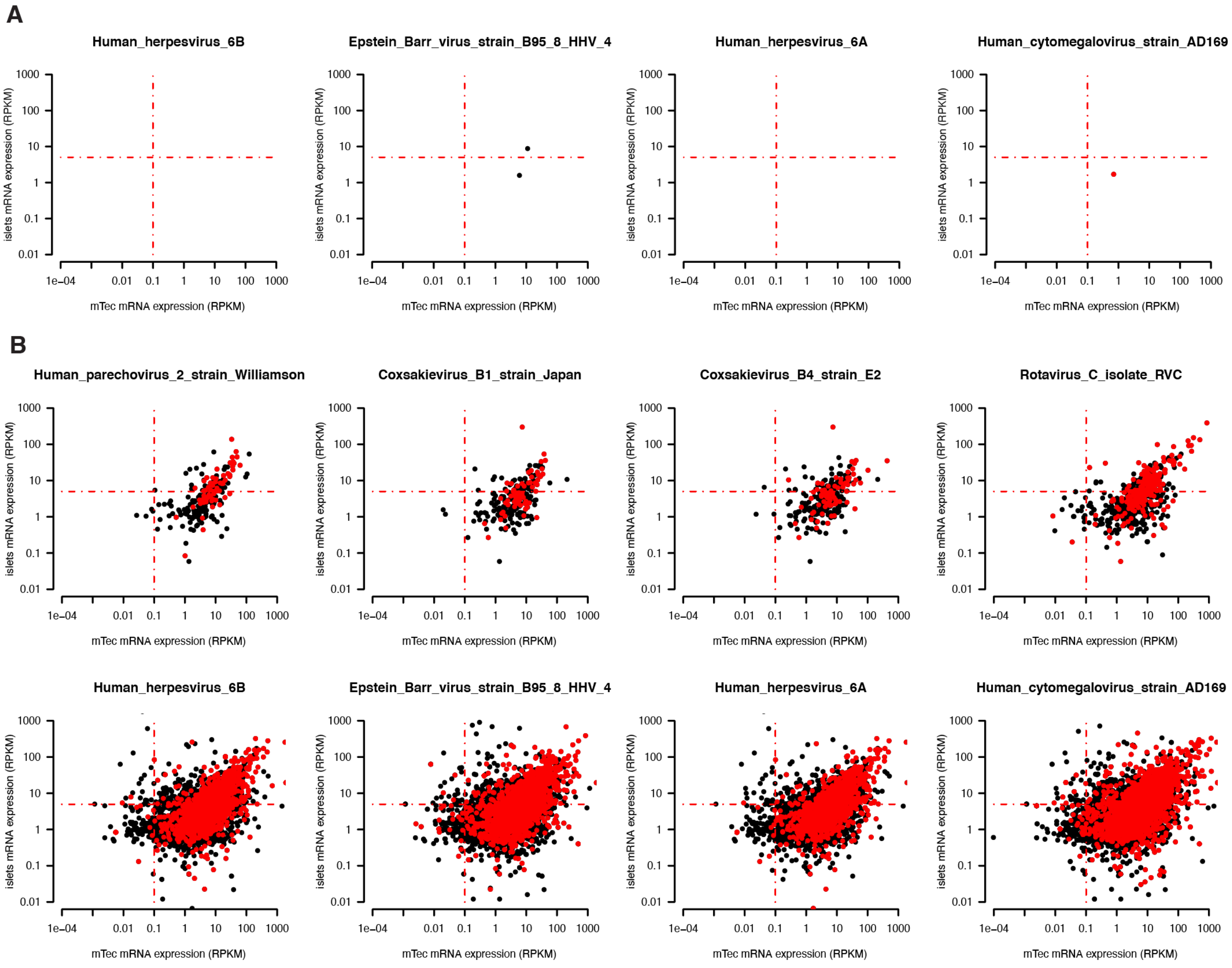

Figure S11. Human pancreatic islets and mTECs' mRNA expression of antigens potentially carrying HLA-B*39:06-restricted viral-human non-spliced and *cis*-spliced *zwitter* peptide candidates. The scatter plots depict the distribution of RPKM of mRNA of human antigens, as measured by Gonzalez-Duque and colleagues [2] in human pancreatic islets and mTECs, that theoretically can carry (**A**) non-spliced and (**B**) *cis*-spliced viral-human *zwitter* epitope candidates. Scatter plots are divided based on the corresponding theoretical virus origin. In (**A**) only four out of eight viruses are shown because for four viruses no viral-human non-spliced peptide candidates with the required characteristics were estimated. Black dots represent antigens carrying epitope candidates predicted to bind the HLA-B*39:06 allele. Red dots represent antigens carrying epitope candidates predicted to bind the HLA-B*39:06 allele and located in hotspots, according to IEDB database.

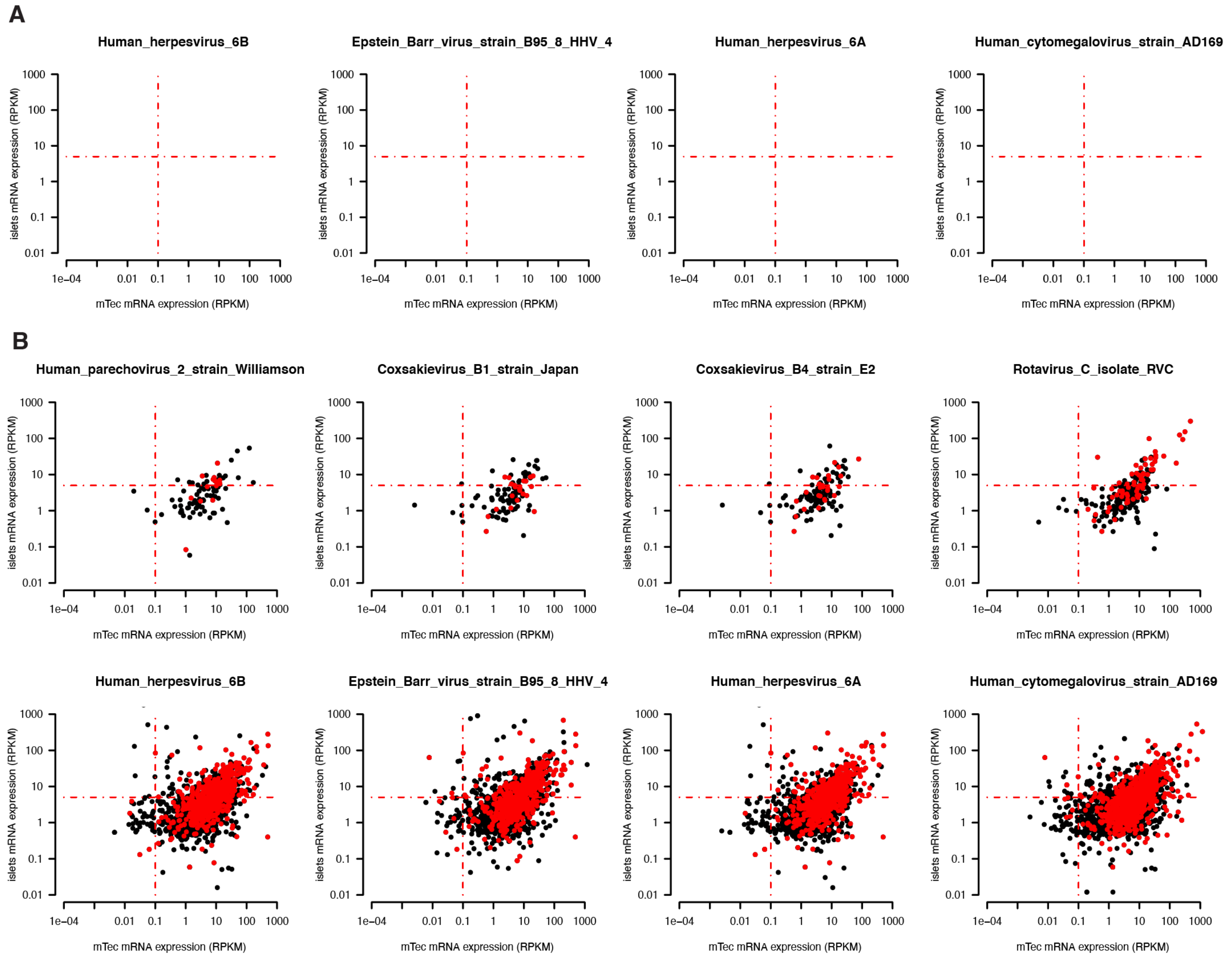

Figure S12. Human pancreatic islets and mTECs' mRNA expression of antigens potentially carrying HLA-B*40:01-restricted viral-human non-spliced and *cis*-spliced *zwitter* peptide candidates. The scatter plots depict the distribution of RPKM of mRNA of human antigens, as measured by Gonzalez-Duque and colleagues [2] in human pancreatic islets and mTECs, that theoretically can carry (**A**) non-spliced and (**B**) *cis*-spliced viral-human *zwitter* epitope candidates. Scatter plots are divided based on the corresponding theoretical virus origin. In (**A**) only four out of eight viruses are shown because for four viruses no viral-human non-spliced peptide candidates with the required characteristics were estimated. Black dots represent antigens carrying epitope candidates predicted to bind the HLA-B*40:01 allele. Red dots represent antigens carrying epitope candidates predicted to bind the HLA-B*40:01 allele and located in hotspots, according to IEDB database.

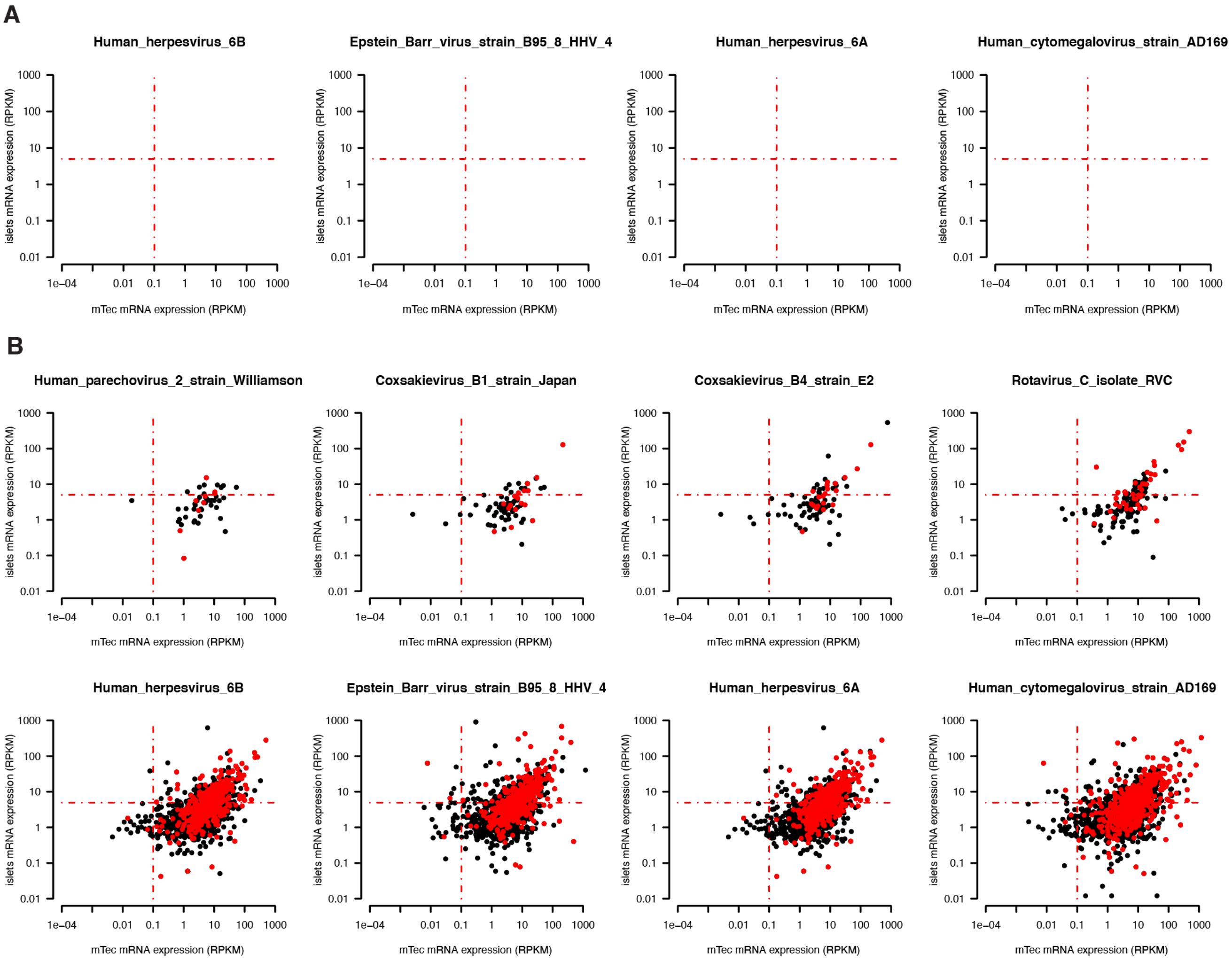

Figure S13. Human pancreatic islets and mTECs' mRNA expression of antigens potentially carrying HLA-B*44:02-restricted viral-human non-spliced and *cis*-spliced *zwitter* peptide candidates. The scatter plots depict the distribution of RPKM of mRNA of human antigens, as measured by Gonzalez-Duque and colleagues [2] in human pancreatic islets and mTECs, that theoretically can carry (**A**) non-spliced and (**B**) *cis*-spliced viral-human *zwitter* epitope candidates. Scatter plots are divided based on the corresponding theoretical virus origin. In (**A**) only four out of eight viruses are shown because for four viruses no viral-human non-spliced peptide candidates with the required characteristics were estimated. Black dots represent antigens carrying epitope candidates predicted to bind the HLA-B*44:02 allele. Red dots represent antigens carrying epitope candidates predicted to bind the HLA-B*44:02 allele and located in hotspots, according to IEDB database.

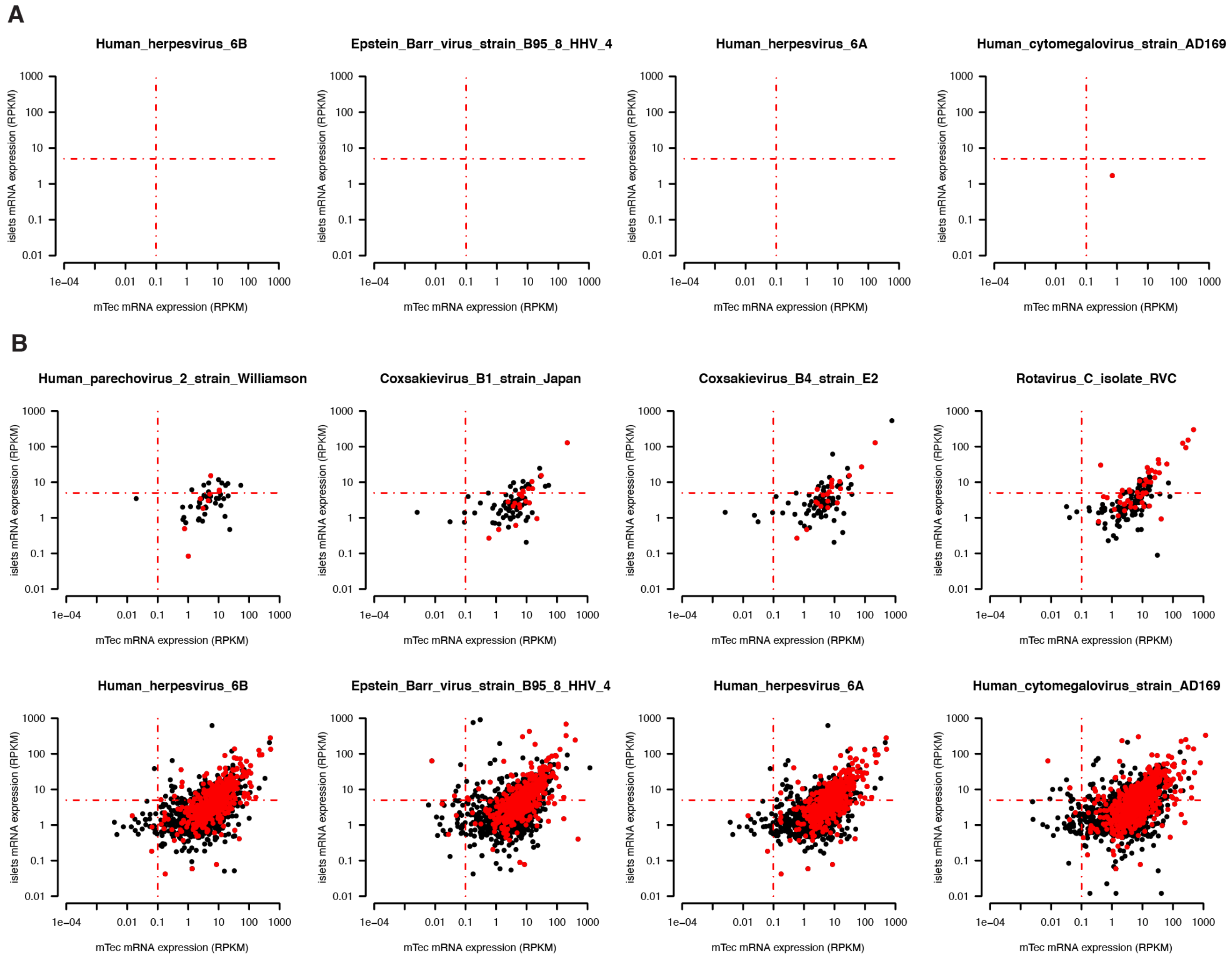

Figure S14. Human pancreatic islets and mTECs' mRNA expression of antigens potentially carrying HLA-B*44:03-restricted viral-human non-spliced and *cis*-spliced *zwitter* peptide candidates. The scatter plots depict the distribution of RPKM of mRNA of human antigens, as measured by Gonzalez-Duque and colleagues [2] in human pancreatic islets and mTECs, that theoretically can carry (**A**) non-spliced and (**B**) *cis*-spliced viral-human *zwitter* epitope candidates. Scatter plots are divided based on the corresponding theoretical virus origin. In (**A**) only four out of eight viruses are shown because for four viruses no viral-human non-spliced peptide candidates with the required characteristics were estimated. Black dots represent antigens carrying epitope candidates predicted to bind the HLA-B*44:03 allele. Red dots represent antigens carrying epitope candidates predicted to bind the HLA-B*44:03 allele and located in hotspots, according to IEDB database.

References

[1] R. Vita, S. Mahajan, J.A. Overton, S.K. Dhanda, S. Martini, J.R. Cantrell, D.K. Wheeler, A. Sette, and B. Peters, The Immune Epitope Database (IEDB): 2018 update. Nucleic Acids Res 47 (2019) D339-D343.

[2] S. Gonzalez-Duque, M.E. Azoury, M.L. Colli, G. Afonso, J.V. Turatsinze, L. Nigi, A.I. Lalanne, G. Sebastiani, A. Carre, S. Pinto, S. Culina, N. Corcos, M. Bugliani, P. Marchetti, M. Armanet, M. Diedisheim, B. Kyewski, L.M. Steinmetz, S. Buus, S. You, D. Dubois-Laforgue, E. Larger, J.P. Beressi, G. Bruno, F. Dotta, R. Scharfmann, D.L. Eizirik, Y. Verdier, J. Vinh, and R. Mallone, Conventional and Neo-Antigenic Peptides Presented by beta Cells Are Targeted by Circulating Naive CD8+ T Cells in Type 1 Diabetic and Healthy Donors. Cell Metab (2018).

[3] V. Jurtz, S. Paul, M. Andreatta, P. Marcatili, B. Peters, and M. Nielsen, NetMHCpan-4.0: Improved Peptide-MHC Class I Interaction Predictions Integrating Eluted Ligand and Peptide Binding Affinity Data. J Immunol 199 (2017) 3360-3368.
